# Loss of FAM60A disrupts Sin3/HDAC control of the Hippo signaling and promotes oncogenic YAP1 activation

**DOI:** 10.1038/s41419-026-08778-y

**Published:** 2026-04-27

**Authors:** Mojnu Miah, Md Nazmul Huda, Md Rafikul Islam, Dayebgadoh Gerald, Sneha Khator, Akash Saha, Jahangir Alam, Janet L. Thornton, Allen Gies, Michael A. Bauer, Kimberly E. Stephens, Bolni M. Nagalo, Mohammad A. Rahman, Michael P. Washburn, Sayem Miah

**Affiliations:** 1 Department of Biochemistry and Molecular Biology, University of Arkansas for Medical Sciences, Little Rock, AR USA; 2 Winthrop P. Rockefeller Cancer Institute, University of Arkansas for Medical Sciences, Little Rock, AR USA; 3 Stowers Institute for Medical Research, Kansas City, MO USA; 4 Department of Cancer Biology, University of Kansas Medical Center, Kansas City, KS USA; 5 Department of Biomedical Informatics, University of Arkansas for Medical Sciences, Little Rock, AR USA; 6 Department of Neuroscience, University of Arkansas for Medical Sciences, Little Rock, AR USA; 7 Department of Pathology, University of Arkansas for Medical Sciences, Little Rock, AR USA; 8 Present Address: Department of Pathology, University of Texas Medical Branch, Galveston, TX USA

**Keywords:** Cell biology, Cancer

## Abstract

FAM60A (also known as SINHCAF) is a subunit of the Sin3/HDAC histone deacetylase complex with established roles in chromatin remodeling, yet its broader cellular functions remain largely undefined. Using immunological, biochemical, CRISPR/Cas9, genomic, and proteomic approaches, we mapped the FAM60A interaction network and its functional impact. We reveal that FAM60A binds directly to HDAC1 to recruit Sin3/HDAC, while a dual-domain architecture mediates additional associations with RNA and DNA-binding proteins. CRISPR/Cas9–mediated HDAC1 knockout abolishes the FAM60A–SIN3A interaction, confirming this dependency. Loss of FAM60A triggers widespread transcriptional rewiring, including downregulation of WWC3—a scaffold for LATS1/2 activation. Consequently, YAP1 dephosphorylation and nuclear accumulation shifted cell-cycle dynamics toward G₁ enrichment and conferred resistance to metabolic stress. Restoration of FAM60A or exogenous WWC3 reactivated Hippo “off” signaling, normalized cell-cycle distribution, and reversed stress resistance. These findings establish FAM60A as a pivotal epigenetic tuner linking histone deacetylation to Hippo pathway regulation and nominate the FAM60A–HDAC1–WWC3 axis as a potential therapeutic target to restore growth control in YAP-driven cancers.

## Introduction

Chromatin remodeling complexes, such as the Sin3/Histone Deacetylase (HDAC) complex, are essential regulators of chromatin structure and accessibility, influencing transcription, replication, and DNA repair [1–4]. The Sin3/HDAC complex facilitates histone deacetylation, resulting in a more compact chromatin state that modulates gene expression programs critical for cellular identity [5, 6]. Although frequently associated with transcriptional repression, genome-wide studies have demonstrated that SIN3A can also function in transcriptional activation, highlighting its context-dependent regulatory roles in gene expression [7–11].

Within this complex, FAM60A (also known as SINHCAF) has emerged as a notable subunit [12]. Proteomic and biochemical studies have consistently identified FAM60A as part of the Sin3/HDAC complex, where it contributes to targeting Sin3/HDAC to specific genomic loci [13, 14]. FAM60A is broadly expressed across mammalian tissues and has been implicated in essential developmental processes, including maintenance of transcriptional homeostasis and promoter methylation balance, with its genetic deletion resulting in widespread developmental defects and embryonic lethality [14, 15]. Despite these insights, the broader molecular functions of FAM60A remain incompletely understood.

Recent evidence suggests that components of chromatin-modifying complexes can interface with signaling networks to influence cellular growth programs, including the Hippo pathway, which maintains tissue homeostasis by restricting YAP/TAZ transcriptional activity [2]. WWC3, in particular, functions as a scaffold for LATS1/2 activation and is required to maintain Hippo “off” signaling by promoting YAP phosphorylation and cytoplasmic retention [16, 17]. Given the importance of WWC3–LATS1/2 signaling in controlling proliferation and cancer progression, understanding how chromatin regulators influence this axis is essential.

In this study, we employ integrated immunological, biochemical, genomic, proteomic, and CRISPR/Cas9 approaches to dissect the roles of FAM60A beyond its canonical association with the Sin3/HDAC complex. We reveal that FAM60A engages the Sin3/HDAC complex through direct interaction with HDAC1, rather than SIN3A itself. Furthermore, we demonstrate that FAM60A’s dual-domain architecture enables additional interactions with RNA- and DNA-binding proteins, expanding its functional repertoire. CRISPR/Cas9-mediated deletion of HDAC1 confirms that SIN3A recruitment is dependent on HDAC1. Functionally, the loss of FAM60A disrupts transcriptional equilibrium, downregulates WWC3, and reprograms Hippo signaling toward enhanced YAP1 nuclear activity, leading to altered cell-cycle distribution and increased proliferative capacity.

Together, our findings position FAM60A as an epigenetic tuner linking histone deacetylation to Hippo pathway control, revealing new mechanistic insights into chromatin remodeling, signal transduction, and cancer biology.

## Results

### FAM60A is a core component of the Sin3/HDAC transcriptional repressor complex

**Fig. 1:**
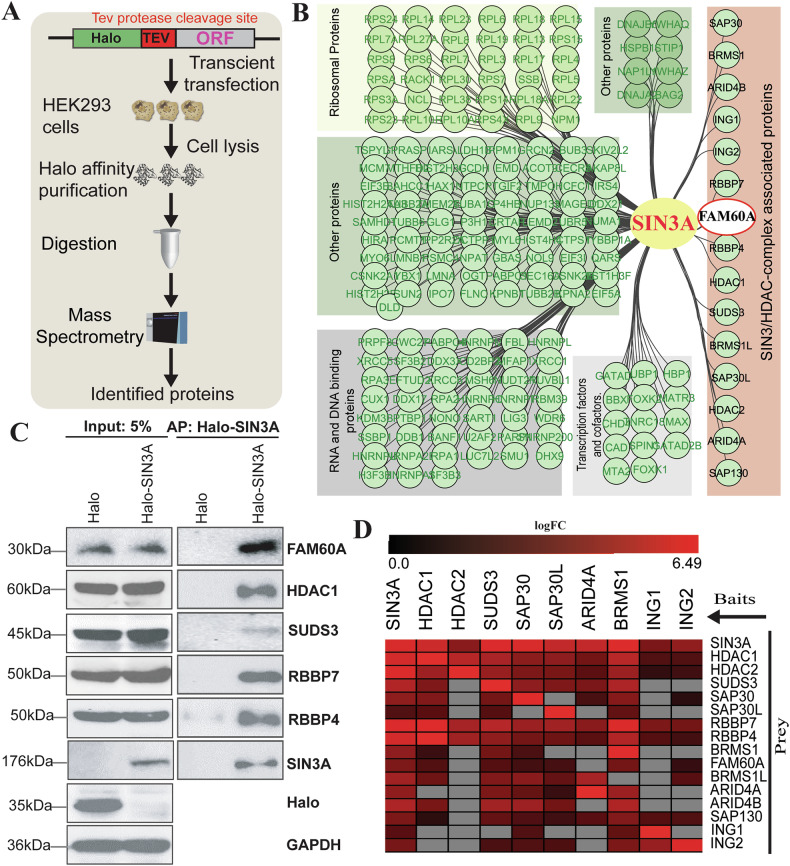
SIN3A network reveals FAM60A as a subunit of Sin3/HDAC. **A** A schematic of a refined proteomics workflow employing Mass spectrometry for protein identification. **B** HEK293T cells were transfected with Halo-SIN3A, and the cell lysates were subjected to affinity purification with Halo magnetic beads. Purified protein complexes were identified by Mass Spectrometry and analyzed using QSPEC (QSPEC log2FC ≥ 1, QSPEC FDR < 0.05). The results were then visualized in a differential network using Cytoscape. **C** Halo-SIN3A or Halo plasmid alone was ectopically expressed in HEK293T cells, followed by Halo affinity purification. The Halo affinity-purified proteins were subjected to immunoblotting with anti-FAM60A, anti-HDAC1, anti-SUDS3, anti-RBBP7, anti-RBBP4, and anti-SIN3A antibodies. Total cell lysates were also analyzed by immunoblotting using the same antibodies and anti-GAPDH as a loading control. **D** Identified subunits of Sin3/HDAC complex were N-terminally Halo-tagged and ectopically expressed in HEK293 cells, followed by affinity purification and identified using Mass-spectrometry analysis (QSPEC log2FC ≥ 1, FDR < 0.05) and visualized in a heatmap.

Earlier research highlighted FAM60A co-purified with several Sin3/HDAC complex members, including BRMS1/1 L, SAP30/30 L, and ING1/2 [12, 18, 19]. To elucidate the interaction network among these proteins and discern relationships between the core subunits of the Sin3/HDAC complex and FAM60A, we integrated the Halo affinity tag with Multidimensional Protein Identification Technology (MudPIT). This approach (Fig. 1A) was validated by expressing Halo-SIN3A in HEK293 cells, purifying the associated protein complexes, quantifying them using MudPIT mass spectrometry, and visualizing them in Cytoscape [20] (Fig. 1B, Supplementary Table 1). Notably, endogenous FAM60A co-purified with Halo-SIN3A alongside other Sin3/HDAC complex subunits such as HDAC1, HDAC2, SUDS3, SAP30, SAP30L, BRMS1, ING1, and ING2 (Fig. 1B, highlighted in red). The interaction of Sin3/HDAC complex subunits with SIN3A was further corroborated through immunoblotting. After expressing Halo-SIN3A in HEK293 cells, we affinity-purified SIN3A-associated proteins, performed SDS PAGE-western blotting, and probed them with antibodies targeting core Sin3/HDAC complex subunits, including FAM60A (Fig. 1C). To confirm FAM60A as a core component of SIN3A-containing complexes, we ectopically expressed Halo-tagged versions of key Sin3/HDAC subunits, including SIN3A, HDAC1, HDAC2, SUDS3, SAP30, SAP30L, ARID4A, BRMS1, ING1, and ING2. Subsequent affinity purification and quantitative MudPIT analysis revealed co-purification of FAM60A with HDAC1 and other subunits, whereas HDAC2 showed only marginal enrichment that did not surpass the applied (log₂FC > 1) thresholds (Fig. 1D, Supplementary Table 1). This pattern is consistent with prior reports demonstrating preferential association of FAM60A with HDAC1, and indicates that HDAC2 interaction, while not absent, occurs at lower affinity or with reduced stoichiometric occupancy relative to HDAC1 within the Sin3/HDAC complex [21]. We observed that endogenous FAM60A co-purified with all core subunits of this complex, except ING1, ARID4A, and HDAC2. It is to note that ARID4A, a transcription factor co-purifying with SIN3A, is not traditionally considered a Sin3/HDAC complex subunit.

Next, to determine what domain/region of SIN3A might interact with FAM60A, we generated a series of deletion mutants. Since SIN3A is a scaffolding protein, we systematically deleted highly conserved regions annotated as specific domains (Supplementary Fig. 1A): four Paired Amphipathic Helices, (PAH1-4); SUDS3 and SAP130 interacting domain; HDAC Interacting Domain (HID); Highly Conserved Region, (HCR) at the C-terminus; 7 poly glutamic acids domain; and Proline-rich domain, however, SIN3A lacks enzymatic activities and DNA binding motifs [8]. Full-length and deletion variants of Halo-tagged SIN3A were overexpressed in HEK293T cells to assess their expression and intracellular localization. We used live-cell imaging to assess the localization of ectopically expressed Halo-SIN3A and Halo-SIN3A deletion mutants by transfecting HEK293T cells and imaging by confocal microscopy (Supplementary Fig. 1B). We observed that Halo-SIN3A and all deletion mutants predominantly localized to the nucleus, while Halo-SIN3A_Δ8, which lacks the 7 poly-Glu domain, mis-localized to the cytosol (Supplementary Fig. 1B).

To identify the essential domains of SIN3A for FAM60A binding, we conducted advanced proteomic analysis on Halo-SIN3A deletion mutants. Halo-tagged SIN3A deletion mutants were ectopically expressed, purified using Halo affinity purification, and then analyzed with liquid chromatography-mass spectrometry (LC-MS) to identify proteins interacting with the deletion mutants. The identified interacting proteins of each Halo-SIN3A deletion mutant are presented in a heatmap (Supplementary Fig. 1C, Supplementary Table 1). We observed that SIN3A and its deletion mutants interact with hundreds of proteins, including the core subunit of the Sin3/HDAC complex (Supplementary Fig. 1C). We then focused on the core subunits of the Sin3/HDAC complex and found that FAM60A did not interact with SIN3A lacking the highly conserved region (HCR) (SIN3A_Δ7; AAs 968–1186), along with BRMS1 and ING1 (Supplementary Fig. 1D, Supplementary Table 1). Moreover, FAM60A displayed reduced interaction with both SIN3A_Δ5 and SIN3A_Δ6 compared with full-length SIN3A. Quantitative mass spectrometry confirmed that all SIN3A mutants were expressed at comparable levels under equal transfection conditions (Supplementary Fig. 1D, top panel). However, immunofluorescence imaging showed lower signal intensity for SIN3A_Δ5 (Supplementary Fig. 1B), which may reflect differences in epitope accessibility or detection efficiency rather than expression level. However, unlike FAM60A, BRMS1 did not interact with SIN3A_Δ6, SIN3A_Δ7, SIN3A_Δ8, while ING1 did not interact with SIN3A_Δ4, SIN3A_Δ5, SIN3A_Δ6, SIN3A_Δ7, and SIN3A_Δ8. We also noticed that SUDS3 did not interact with SIN3A_Δ5, and SAP130 did not interact with SIN3A_Δ3 mutants. We also identified domain-specific interactions between SIN3A and various transcription factors, including ARID4A, ARID4B, and MAX (Supplementary Fig. 1D). Overall, our data suggest that FAM60A is a core component of the Sin3/HDAC complex and binds to the C-terminal HCR region of SIN3A.

### Diverse protein interaction network of FAM60A extends beyond the Sin3/HDAC complex

**Fig. 2:**
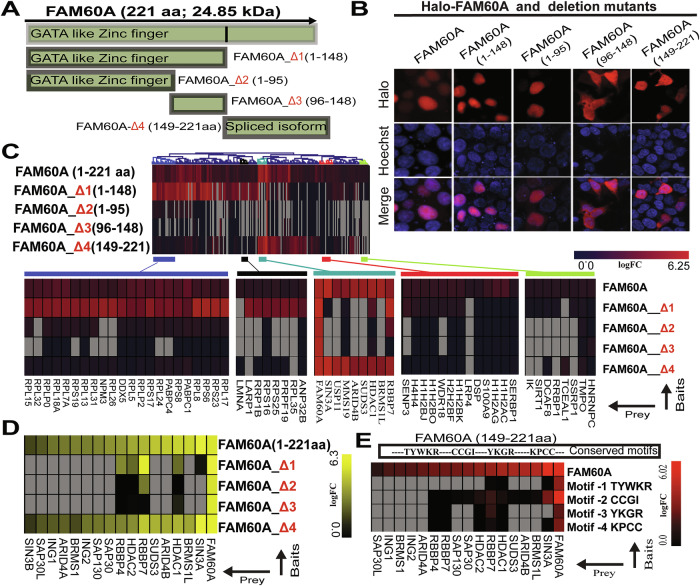
Enhanced deletion analysis of the FAM60A Protein. **A** Schematic diagram illustrates various deletions in the *FAM60A* gene. **B** Halo-tagged FAM60A deletion mutants were transfected into HEK293T cells, labeled with Halo-Tag TMRDirect fluorescent ligand (red), and DNA was stained using Hoechst dye (blue). **C** Halo-FAM60A deletion mutants were expressed in HEK293T cells, purified, and analyzed via proteomics (QSPEC log2FC ≥ 1, FDR < 0.05). Results were displayed in a heatmap. **D** The interactions of full-length and truncated FAM60A with Sin3/HDAC complex core-subunits were visualized in a heatmap (grey indicates no interaction). **E** Heatmap showing FAM60A conserved motif-specific interactions with Sin3/HDAC complex core-subunits (Grey signify no interaction).

FAM60A is a highly conserved protein across many vertebrates (Supplementary Fig. 2A), suggesting its important role in physiological processes. To uncover FAM60A’s biological functions, we generated a series of Halo-tagged FAM60A deletion mutants (Fig. 2A). To assess the proper expression and localization of Halo-FAM60A deletion mutants, we overexpressed them in HEK293T cells and visualized their localization using live-cell imaging (Fig. 2B). Additionally, we performed Halo affinity purification followed by gel electrophoresis and silver staining to visualize the affinity-purified proteins (Supplementary Fig. 2B). Subcellular localization analysis revealed that the full-length Halo-FAM60A, along with its deletion mutants FAM60A_Δ1 (consisting of amino acids (AAs) 1–148), FAM60A_Δ2 (only containing the GATA like zinc finger domain, AAs 1-95), and the spliced isoform FAM60A_Δ4 (only containing the C-terminal AAs 149-221), were localized to the nucleus, while mutant FAM60A_Δ3 (consisting of AAs 96-148 only) exhibited non-nuclear localization (Fig. 2B). This observation aligns with our silver staining analysis, where FAM60A_Δ3 did not interact with other proteins (Supplementary Fig. 2B), suggesting potential instability or improper expression of this truncated protein fragment.

Next, FAM60A and its mutants were affinity-purified and analyzed by proteomics. We observed that FAM60A and its four mutants interacted with hundreds of proteins, which are presented in a heatmap generated with Genesis [22, 23] (Fig. 2C, Supplementary Table 2). Examination of the interaction landscape (Supplementary Fig. 3A) revealed that full-length FAM60A and FAM60A_Δ4 (AAs 149–221) preferentially associated with components of the Sin3/HDAC complex, whereas FAM60A_Δ1 (AAs 1–148) displayed a broader interaction profile. Although the FAM60A_Δ1 pulldown showed higher enrichment of several proteins with known RNA-associated functions, this enrichment may reflect properties of the isolated N-terminal segment, which could expose interaction surfaces not accessible in the full-length protein or alter its folding, thereby increasing susceptibility to indirect or nonphysiological associations. Consistent with this more conservative interpretation, immunoblotting of core Sin3/HDAC subunits confirmed robust interactions with full-length FAM60A and FAM60A_Δ4, aligning with our AP-MS results (Supplementary Fig. 3B).

FAM60A predominantly interacts with the core elements of the Sin3/HDAC complex via its C-terminus. Yet, the submodule consisting of HDAC1, HDAC2, RBBP4, and RBBP7 interacted with both the N- and C-terminus regions of FAM60A (Fig. 2D, Supplementary Table 2, Supplementary Fig. 3A). Since FAM60A predominantly interacts with the Sin3/HDAC complex via its C-terminus, we examined four conserved amino acid motifs located in the C-terminus of FAM60A to dissect better this interaction (Supplementary Fig. 3C).

The motifs in FAM60A were meticulously altered by substituting amino acids with structurally similar counterparts, such as replacing tyrosine (Y) with phenylalanine (F), to minimize the impact on structure and function while preserving the protein’s overall integrity. These modified versions of FAM60A were then introduced into HEK293 cells. Following affinity purification and proteomic analysis, it became evident that Motif-1 (TYWKR) is crucial for FAM60A interaction with the Sin3/HDAC complex, while Motif-3 and 4 also play significant roles. However, Motif-2 did not appear to be important in this interaction (Fig. 2E, Supplementary Table 2).

### HDAC1 mediates FAM60A association with the Sin3/HDAC complex

**Fig. 3:**
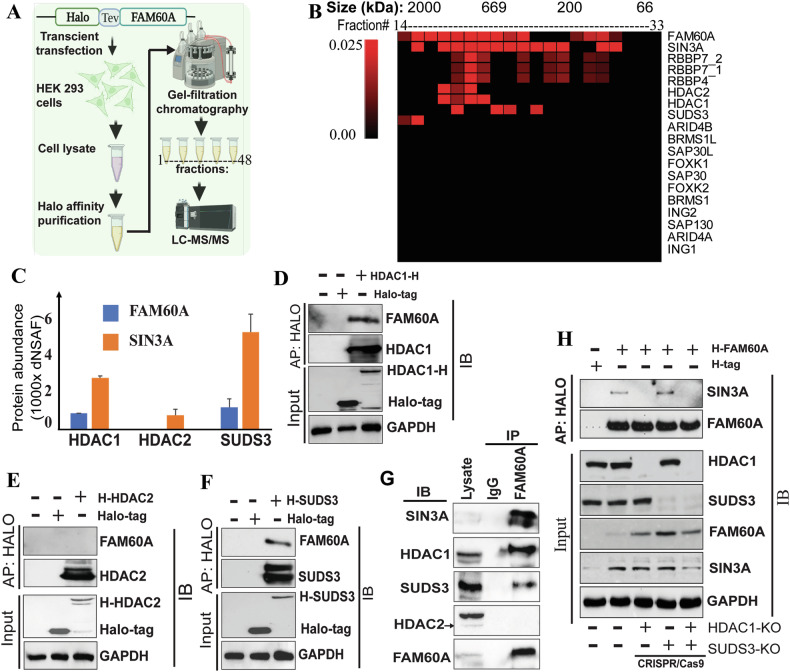
Architecture of the FAM60A-SIN3A/HDAC1 Complex. **A** Workflow for Halo-FAM60A-expressing HEK293 cell extract preparation, Halo-Tag protein purification, size exclusion chromatography, and AP-MS analysis. **B** Halo-FAM60A affinity-purified proteins were fractionated through size-exclusion chromatography and analyzed by LC-MS and visualized in a heatmap (Red and Black). **C** HDAC1-Halo or Halo-HDAC2, or Halo-SUDS3 was ectopically expressed in HEK293T cells. Halo affinity purification followed by mass spectrometry analysis showed that FAM60A and SIN3A copurified with HDAC1-Halo and Halo-SUDS3 but not with Halo-HDAC2. **D**–**F** HDAC1-Halo or Halo-HDAC2, or Halo-SUDS3 or Halo plasmid alone, was ectopically expressed in HEK293T cells. Halo affinity purification was performed, followed by immunoblot analysis using specific antibodies and GAPDH as a loading control. **G** Whole-cell lysates from HEK293 cells were subjected to immunoprecipitation using an anti-FAM60A antibody, followed by immunoblotting with antibodies against HDAC1, HDAC2, SUDS3, and Sin3A. **H** *HDAC1* and *SUDS3* were individually or combinatorially knocked out using CRISPR/Cas9. Subsequently, either the Halo tag alone or Halo-FAM60A was overexpressed, and protein complexes were purified by affinity purification. These complexes were then subjected to immunoblotting analysis and probed with various antibodies.

To address the challenges in identifying the distinct multi-subunit protein complexes and their core components that may interact with FAM60A, we further fractionated the eluate obtained from the Halo-FAM60A affinity purification by size exclusion chromatography (Fig. 3A). We specifically processed the affinity-purified FAM60A by loading it onto a Superose 6 column and collecting 48 fractions of 500 μl each. From these, 20 fractions (14-33) were selected, digested, and then analyzed using MudPIT. Although FAM60A’s expected molecular weight is 29 kDa, its fractionation profile confirmed that it assembles into larger complexes. To elucidate the molecular composition of potential FAM60A-Sin3/HDAC complexes, we assessed whether components of this complex were present. Our observations revealed that FAM60A co-fractionated with SIN3A, RBBP7, RBBP4, HDAC1, HDAC2, and SUDS3 (Fig. 3B, Supplementary Table 3).

Although SIN3A, RBBP7, RBBP4, HDAC1, HDAC2, and SUDS3 co-fractionate with FAM60A, RBBP4 and RBBP7 function as core components of the NuRD complex [24]. To determine specific interactions within the Sin3/HDAC complex, we overexpressed Halo-tagged HDAC1, HDAC2, and SUDS3, performed affinity purification, and analyzed the co-purified proteins. FAM60A is specifically co-purified with HDAC1 and SUDS3 but not with HDAC2 (Fig. 3C). Immunoblotting further validated these findings, showing that ectopically expressed Halo-tagged HDAC1 and SUDS3 interacted with endogenous FAM60A (Fig. 3D, F), whereas HDAC2 failed to do so (Fig. 3E). To determine whether endogenous FAM60A behaves similarly under native expression conditions, we performed immunoprecipitation (IP) using an anti-FAM60A antibody and observed the same interaction pattern, with robust association of FAM60A with HDAC1 and SUDS3 but not with HDAC2 (Fig. 3G).

Previously, we reported that SIN3A crosslinked with both HDAC1 and SUDS3 but not with FAM60A [19, 25]. To investigate whether SUDS3 and HDAC1 mediate the FAM60A-SIN3A interaction, we generated HEK293 knockout cells lacking SUDS3, HDAC1, or both (Fig. 3H). After knockout, we ectopically expressed Halo-FAM60A, performed affinity purification, and probed for SIN3A using immunoblotting. The results showed that the FAM60A-SIN3A interaction was lost in HDAC1-knockout cells but remained intact in SUDS3-knockout cells (Fig. 3H). These findings demonstrate that HDAC1, but not SUDS3 or HDAC2, facilitates the association of FAM60A with the Sin3/HDAC complex, providing mechanistic insight into its molecular interactions.

### FAM60A knockout disrupts transcriptional homeostasis and alters Hippo signaling

**Fig. 4:**
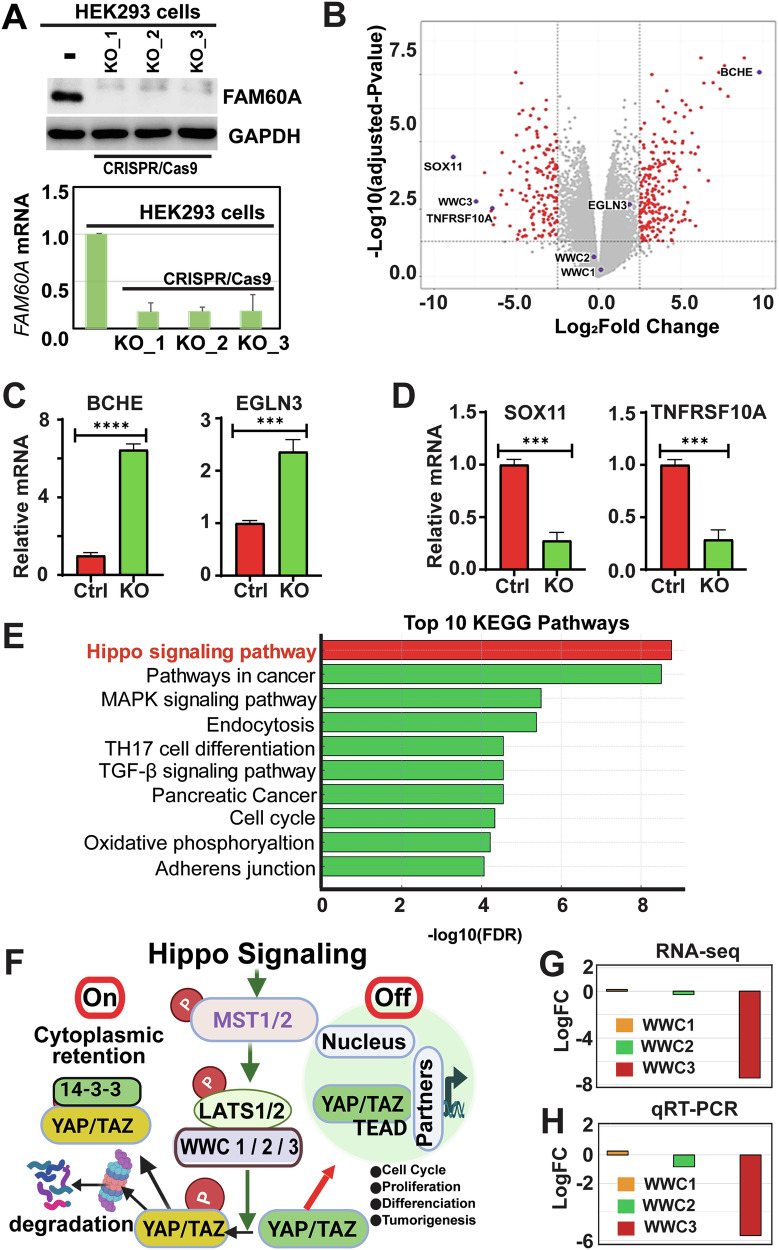
CRISPR-mediated depletion of FAM60A in HEK293 cells reveals altered gene expression associated with the Hippo signaling pathway. **A** Multi-guide-CRISPR/Cas9 was employed to knock out (KO) FAM60A in HEK293 cells. Lysates from FAM60A KO cells were analyzed by immunoblotting using antibodies against FAM60A and GAPDH as a loading control. Gene knockout was also confirmed by quantitative real-time PCR (qRT-PCR). **B** Volcano plot illustrates the differential gene expression between HEK293 control cells and FAM60A knockout cells, as determined by RNA-seq. Red dots represent significantly up- or down-regulated genes (log2FC ≥ 2.5, FDR ≤ 0.05); grey dots indicate genes without significant expression changes. **C**, **D** qRT-PCR was conducted to examine at least two upregulated and two downregulated genes from the RNA-seq data of the HEK293 control and FAM60A knockout cells. ***P ≤ 0.001. **E** KEGG pathway analysis was performed on the significantly up- and down-regulated genes filtered by FDR ≤ 0.05 and log2(FC) > 2.5 between FAM60A knockout and control cells, and the top 10 significant pathways associated with these differentially regulated genes are presented in a bar diagram. **F** Schematic diagram illustrating canonical activation and inactivation of the Hippo signal transduction pathway. **G** RNA-seq analysis revealed significant downregulation of WWC3 in FAM60A-knockout cells compared with control cells. **H** qRT-PCR analysis revealed significant downregulation of WWC3 in the FAM60A knockout cells in comparison to the control cells.

Given the role of FAM60A in chromatin regulation, we next investigated how FAM60A influences gene expression. To further explore its function, we generated FAM60A-knockout HEK293 cells using CRISPR, with knockout confirmed by both immunoblotting and qRT-PCR (Fig. 4A; immunoblotting, top; qRT-PCR, bottom). RNA-seq analysis of control and FAM60A-knockout cells revealed that 272 genes were suppressed, while 383 genes were upregulated compared to controls (log2FC ≥ 2, *n* = 3, *P* ≤ 0.05) (Fig. 4B, Supplementary Table 4). These findings were validated by qRT-PCR for selected genes, confirming the upregulation of *BCHE* and *EGLN3* by over 7-fold and 2.5-fold, respectively (Fig. 4C), and the downregulation of *SOX11* and *TNFRSF10A* by 8-fold and 7-fold, aligning with RNA-seq data (Fig. 4D). To determine the broader impact of FAM60A loss, we conducted KEGG pathway analysis on the significantly regulated genes (log2FC ≥ 2.5, *P* ≤ 0.05), identifying 21 significantly affected pathways, with the top 10 presented in Fig. 4E and Supplementary Table 4. Notably, key signaling cascades—including the Hippo, Wnt, and TGF-β pathways—were disrupted upon FAM60A loss. The analysis revealed extensive crosstalk among these pathways, which coordinately regulate the transcription of anti-apoptotic and pro-proliferative genes (Supplementary Fig. 4). This suggests that depletion of FAM60A shifts signaling dynamics toward a state that favors cell survival and uncontrolled proliferation. Among the affected pathways, Hippo signaling was most significantly impacted (Fig. 4E), a pathway essential for controlling organ size, cell proliferation, apoptosis, and stem cell renewal (Fig. 4F). Given the prominent impact of FAM60A loss on Hippo signaling, we further investigated the mechanistic link between FAM60A and this pathway. RNA-seq analysis identified a pronounced ( > 7-fold) suppression of WWC3, a critical scaffold protein for LATS1/2 activation in Hippo signaling, whereas WWC1 and WWC2 remained unchanged (Fig. 4G). This finding was corroborated by qRT-PCR, affirming RNA-seq data (Fig. 4H). These findings underscore the role of FAM60A in preserving transcriptional homeostasis and highlight its broader significance in coordinating cellular regulatory networks.

### FAM60A modulates Hippo pathway activity via interaction with the Sin3/HDAC complex

**Fig. 5:**
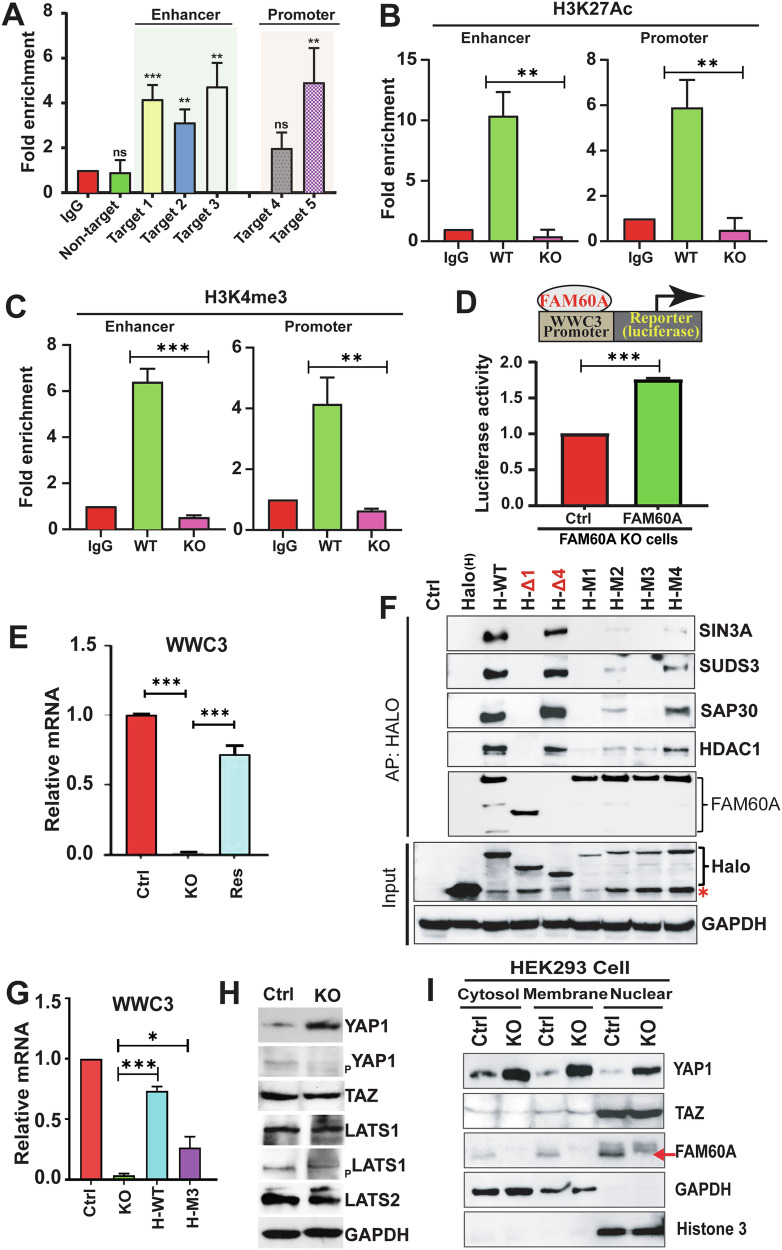
FAM60A controls Hippo signaling through interaction with the Sin3/HDAC complex. **A** ChIP-qPCR analysis revealing FAM60A enrichment at the WWC3 enhancer and promoter regions (*n* = 3, *P ≤ 0.01; ***P ≤ 0.001). **B**, **C** ChIP–qPCR was performed in wild-type (WT) and FAM60A-knockout (KO) cells using antibodies against (b) H3K27ac and (c) H3K4me3. Enrichment at the WWC3 promoter and enhancer regions is shown relative to IgG control (*n* = 3, *P ≤ 0.01; ***P ≤ 0.001). **D** Luciferase reporter assay in HEK293 cells demonstrating that FAM60A enhances WWC3 promoter–driven transcription. **E** Halo-FAM60A was expressed in FAM60A knockout HEK293 cells, and the expression of WWC3 was determined by qRT-PCR (*n* = 3, ***P ≤ 0.001). **F** Halo-FAM60A Wild type (H-WT) and deletion mutants Halo-FAM60A_Δ1: 1–148 (H-Δ1), Halo-FAM60A_Δ4: 149-221(H-Δ4), Halo-tagged FAM60A motif 1–4, were transfected into HEK293 cells, affinity purified, and analyzed by immunoblotting using antibodies against different specific targets, and GAPDH as a loading control. **G** Besides, Halo-FAM60A, Halo-FAM60A motif-3 was expressed in FAM60A knockout HEK293 cells and the expression of WWC3 was determined by qRT-PCR (*n* = 3, *P ≤ 0.05; ***P ≤ 0.001). **H** Protein levels of various Hippo signaling pathway molecules were examined in FAM60A knockout cells and control cells by immunoblotting using antibodies against different specific targets, and GAPDH as a loading control. **I** FAM60A knockout cells and the HEK293 control cells were fractionated into the cytosolic, membrane, and nuclear fractions and subjected to immunoblotting analysis for the detection of YAP1 and TAZ. GAPDH and Histone H3 were used as controls for the cytosolic/membrane and nuclear compartments, respectively. * Indicates non-specific.

Given the pronounced suppression of WWC3 following FAM60A loss, we examined whether FAM60A occupies the WWC3 enhancer and promoter regions. Chromatin immunoprecipitation followed by PCR, guided by prior ChIP-seq studies [26, 27] on Sin3A occupancy at these sites (Supplementary Fig. 5A, Supplementary Fig. 5B), revealed significant enrichment of FAM60A at both the enhancer and promoter regions of WWC3 in wild-type cells (Fig. 5A). To determine whether FAM60A loss alters the chromatin state at the WWC3 locus, we performed ChIP–qPCR for active chromatin marks H3K27ac and H3K4me3. In wild-type cells, both marks were significantly enriched at the WWC3 promoter and enhancer regions. In contrast, FAM60A-knockout cells exhibited markedly reduced enrichment of H3K27ac (Fig. 5B) and H3K4me3 (Fig. 5C), indicating a loss of active chromatin configuration at these regulatory elements. Consistent with these chromatin changes, a luciferase reporter assay demonstrated that FAM60A significantly enhances WWC3 promoter activity (Fig. 5D), and reintroduction of wild-type FAM60A into knockout cells restored WWC3 expression by approximately 70% (Fig. 5E).

Next, we interrogated whether Sin3/HDAC binding is required for this regulation. To test that, we generated several FAM60A deletion and motif mutants centered on the C-terminal region, which we established as essential for Sin3/HDAC binding, as detailed in Fig. 2 and Supplementary Fig. 3. Affinity purification followed by immunoblotting analysis demonstrated that mutants lacking the C-terminal region (FAM60A_Δ1: 1–148) or specific critical motifs (e.g., H-M1, H-M3) failed to interact with the Sin3/HDAC complex, while mutants maintaining intact C-terminal motifs (H-Δ4), and motif mutants (H-M2, H-M4) retained binding capability (Fig. 5F). Notably, mutant H-M3, despite a reduced capacity to interact with the Sin3/HDAC complex, partially restored WWC3 expression (~20%) compared to wild-type rescue (~70%), highlighting the functional importance of the interaction of FAM60A with the Sin3/HDAC complex in regulating WWC3 expression (Fig. 5G, Supplementary Fig. 5B). Collectively, these observations establish a clear mechanistic link between FAM60A, the Sin3/HDAC complex, and the modulation of Hippo pathway activity.

We next evaluated downstream effectors of the Hippo pathway. Immunoblot analysis revealed substantial upregulation of YAP1 in cells lacking FAM60A, while total levels of TAZ, LATS1, and LATS2 remained unchanged. Phosphorylation of LATS1 was partially attenuated in FAM60A-knockout cells, and phosphorylation of YAP1, indicative of Hippo pathway activation, was markedly reduced (Fig. 5H). To assess subcellular distribution, cellular fractionation followed by immunoblotting demonstrated increased nuclear enrichment of YAP1 in FAM60A-knockout cells, whereas TAZ localization remained unchanged (Fig. 5I). To corroborate the biochemical fractionation results, YAP1 localization was examined by immunocytochemistry. Consistent with the fractionation data, FAM60A-knockout MDA-MB-231 cells exhibited increased nuclear accumulation of YAP1 relative to control cells (Supplementary Fig. 5C). To determine the downstream transcriptional consequences of YAP1 activation, the expression of canonical YAP1 target genes [28] was assessed, revealing robust upregulation of CTGF and CYR61 in FAM60A-knockout cells (Supplementary Fig. 5D). Together, these analyses demonstrate that FAM60A loss reduces YAP1 phosphorylation, promotes its nuclear accumulation, and increases expression of YAP1-responsive genes.

### FAM60A regulates proliferation and cell-cycle dynamics through Hippo signaling

**Fig. 6:**
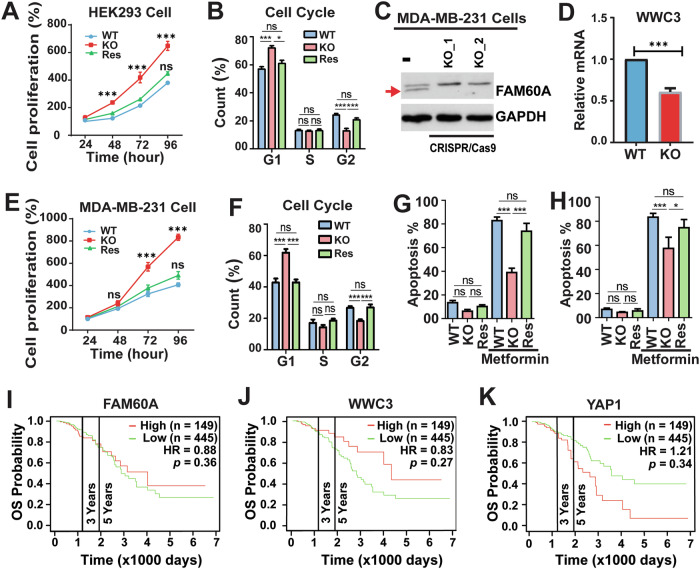
FAM60A drives cell-cycle dynamics and proliferation via regulation of Hippo signaling. **A** Cell proliferation analysis of HEK293 control cells, FAM60A knockout cells, and FAM60A reintroduced in the knockout cells. Cell proliferation was quantified using the WST-1 assay. ***p ≤ 0.001). **B** Cell cycle analysis for the HEK293 control cells, FAM60A knockout cells, and FAM60A reintroduced cells using flow cytometry after staining with propidium iodide (PI). Data are presented as the means ± SD (*n* = 3) of triplicate experiments. *P ≤ 0.05; ***P ≤ 0.001. **C** Multi-guide-CRISPR/Cas9 was employed to knock out (KO) FAM60A in MDA-MB-231 cells, and lysates from FAM60A KO cells were analyzed by immunoblotting using antibodies against FAM60A and GAPDH as a loading control. **D** The expression of WWC3 in FAM60A KO cells and MDA-MB-231 control cells was determined by qRT-PCR (*n* = 3, **P ≤ 0.001). **E** Cell proliferation analysis of MDA-MB-231 control cells, FAM60A knockout cells, generated via CRISPR-Cas9, and FAM60A reintroduced in the knockout cells. Cell proliferation was quantified using the WST-1 assay. ***p ≤ 0.001). **F** Cell cycle analysis for FAM60A knockout cells and the MDA-MB-231 control cells, and FAM60A reintroduced cells using flow cytometry after staining with propidium iodide (PI). Data are presented as the means ± SD of triplicate experiments. *P ≤ 0.05; ***P ≤ 0.001. **G**, **H** Apoptosis analyses in control, FAM60A KO, and FAM60A-reintroduced HEK293 and MDA-MB-231 cells treated with 10 μM metformin for 24 h, evaluated by Annexin V/PI staining by flow cytometry. WT Wild type; KO Knockout; Res: Rescue with FAM60A in KO cells. Mean ± SD (*n* = 3); ***P ≤ 0.001, *P ≤ 0.05. ns: not significant. **I**–**K** Kaplan–Meier survival curves were generated using PROGgene V2 based on TCGA breast cancer datasets, with patients grouped using the 75th percentile expression cutoff. Panels show overall survival stratified by FAM60A (**I**), WWC3 (**J**), and YAP1 (**K**) expression, with corresponding hazard ratios (HR) and *p*-values.

Given the critical role of Hippo signaling in proliferation and apoptosis, we evaluated the cellular consequences of FAM60A depletion. Proliferation assays in HEK293 cells showed significantly increased proliferation upon FAM60A knockout, an effect completely reversed by reintroducing wild-type FAM60A (Fig. 6A, Supplementary Fig. 6A). To substantiate the contribution of WWC3 to this phenotype, ectopic overexpression of FLAG-WWC3 in FAM60A-knockout cells effectively abolished the enhanced proliferation induced by FAM60A loss (Supplementary Fig. 6B). To further define the requirement for downstream Hippo pathway effectors, YAP1 was knocked down in FAM60A-knockout cells, resulting in a marked suppression of cell proliferation, indicating that YAP1 activity is essential for the proliferative advantage conferred by FAM60A depletion (Supplementary Fig. 6C). Collectively, these findings support a model in which FAM60A regulates cell proliferation through coordinated control of WWC3 and YAP1 within the Hippo signaling pathway.

Further analysis of cell-cycle dynamics revealed marked accumulation in the G1 phase and a corresponding reduction in progression to the G2 phase in FAM60A-knockout cells, effects fully restored by reintroducing FAM60A (Fig. 6B, Supplementary Fig. 6D). These observations were reproducible in MDA-MB-231 cells upon FAM60A knockout (Fig. 6C, E, F), accompanied by consistent WWC3 downregulation verified by qRT-PCR (Fig. 6D, Supplementary Fig. 6E, F). Apoptosis assays indicated that FAM60A depletion did not alter baseline apoptotic rates, but markedly decreased cell death induced by metformin in both HEK293 and MDA-MB-231 cells (Fig. 6G, H, Supplementary Fig. 7A, B, respectively). Additional evaluation with the chemotherapeutic agent doxorubicin similarly demonstrated reduced apoptosis in FAM60A-knockout cells in both HEK293 and MDA-MB-231 cell lines, as shown by Annexin V/PI cytometric profiles and corresponding quantitative analyses (Supplementary Fig. 7C, D). Collectively, these data indicate that FAM60A loss confers a survival advantage and alters proliferative and apoptotic responses. To extend these findings to clinical datasets, we analyzed overall survival in TCGA breast cancer cohorts using PROGgene V2 [29]. Kaplan–Meier analysis showed that higher expression of FAM60A (hazard ratio [HR] = 0.88, *p* = 0.36) and WWC3 (HR = 0.83, *p* = 0.27) was associated with a trend toward improved overall survival, whereas elevated YAP1 expression (HR = 1.21, *p* = 0.34) showed a trend toward poorer outcomes (Fig. 6I-K). Although these associations did not reach statistical significance, the directionality of the hazard ratios is consistent with our proposed mechanistic model and provides preliminary clinical context for the FAM60A–WWC3–YAP1 regulatory axis.

## Discussion

FAM60A, an indispensable subunit of the Sin3/HDAC complex, plays a critical role in gene regulation and chromatin remodeling [12, 14, 15]. While its integration into the Sin3/HDAC complex is well-established, FAM60A’s broader cellular functions remain largely unexplored. Notably, FAM60A plays a crucial role in maintaining stem cell pluripotency [14], with its absence resulting in embryonic lethality [15], indicating its broader, yet undefined, roles in cellular systems. Through comprehensive molecular dissection, our study reveals: (i) FAM60A’s integral association with the Sin3/HDAC complex; (ii) a modular domain structure enabling broader protein interactions beyond Sin3/HDAC; (iii) interaction with SIN3A mediated indirectly via HDAC1, revealed through CRISPR/Cas9 mutagenesis; (iv) disruption of transcriptional homeostasis and Hippo pathway reprogramming upon FAM60A loss; and (v) subsequent alterations in proliferation and cell-cycle progression. Collectively, these findings position FAM60A as a pivotal regulator linking chromatin remodeling to proliferative signaling, transcriptional balance, and cellular survival.

FAM60A was initially characterized as a Sin3/HDAC complex subunit based on AP-MS studies[12, 14, 18]. Our AP-MS profiling corroborates these findings, demonstrating reciprocal interactions between FAM60A and SIN3A, along with other Sin3/HDAC components (Fig. 1B–D). Interestingly, FAM60A employs distinct domains to engage different molecular cohorts: its C-terminus for Sin3/HDAC subunits and its N-terminus for RNA and DNA-binding proteins (Fig. 2C, D), underscoring its dual functional capacity. However, AP-MS cannot unequivocally resolve direct versus indirect interactions. SIN3A, as the scaffold of the Sin3/HDAC complex [5, 30], would be expected to bind FAM60A directly. Surprisingly, our combination of gel-filtration chromatography and mutagenesis study revealed that FAM60A does not directly engage SIN3A. Instead, FAM60A is incorporated into the complex through direct binding to HDAC1 (Fig. 3C–F). These findings refine the understanding of the architectural integration of FAM60A within the Sin3/HDAC machinery, identifying HDAC1 as the essential molecular bridge.

The loss of FAM60A induces widespread transcriptional rewiring, consistent with the broad coregulatory roles of Sin3/HDAC complexes in controlling diverse gene networks [7]. RNA-seq profiling revealed dysregulation of 655 genes across 21 pathways, including enrichment in Hippo, Wnt, and TGF-β signaling (Fig. 4E). These pathways converge at multiple levels to govern proliferation, apoptosis, and differentiation [31] (Supplementary Fig. 4). Crosstalk between Hippo and Wnt pathways can synergize to co-activate YAP/β-catenin target genes [32], while TGF-β/Smad interactions dynamically modulate this axis in a context-dependent manner [33]. Given that Sin3/HDAC complexe represses gene expression via promoter deacetylation [34, 35], FAM60A likely serves as a critical epigenetic tuner at key signaling hubs. Its loss may result in hyperacetylation and aberrant activation of oncogenic programs while simultaneously silencing tumor suppressor genes [12, 36, 37], thereby promoting uncontrolled proliferation and survival.

Importantly, our study identifies a functional connection between chromatin remodeling and Hippo pathway regulation mediated by FAM60A. WWC3, a scaffold required for LATS1/2 activation [16], was significantly downregulated following FAM60A depletion, accompanied by increased expression of canonical YAP1–TEAD target genes. Although the Sin3/HDAC complex is classically associated with transcriptional repression, genome-wide analyses have demonstrated context-dependent roles for SIN3A in transcriptional activation [7–11]. Consistent with this framework, ChIP-seq datasets indicate SIN3A occupancy at the WWC3 promoter [26, 27], and our data show that FAM60A-associated Sin3/HDAC complexes are required to maintain WWC3 expression. Loss of FAM60A correlated with reduced WWC3 transcription and diminished active chromatin marks at its regulatory regions, supporting a role for FAM60A in sustaining promoter competence rather than repression (Fig. 5). Reduced WWC3 expression was associated with decreased LATS1 phosphorylation and increased nuclear YAP1 accumulation.

Functionally, FAM60A loss resulted in enhanced proliferation and altered cell-cycle distribution, with accumulation in G₁ phase and reduced progression into G₂/M. These effects were reversed by reintroduction of FAM60A or ectopic expression of WWC3, demonstrating that WWC3 is a critical mediator downstream of FAM60A (Fig. 6). Importantly, silencing of YAP1 in FAM60A-knockout cells completely abrogated the proliferative advantage, establishing that YAP1 activity is required for the growth phenotype associated with FAM60A depletion (Supplementary Fig. 6). In parallel, FAM60A-deficient cells displayed reduced sensitivity to metabolic and chemotherapeutic stress. Together, these findings support a model in which FAM60A maintains WWC3-dependent Hippo signaling to restrain YAP1-driven proliferative and survival programs.

In parallel, FAM60A-deficient cells exhibited resistance to both metformin- and doxorubicin-induced apoptosis [38–40], indicating that loss of FAM60A confers a survival advantage under metabolic and chemotherapeutic stress. Transcriptomic analysis revealed marked downregulation of TNFRSF10A, which encodes Death Receptor 4 (DR4), a TRAIL receptor that mediates extrinsic apoptosis through recruitment of FADD and activation of caspase-8 [41–43]. Although reduced TNFRSF10A expression is consistent with diminished sensitivity to extrinsic apoptosis, our data do not establish a direct causal link between its downregulation and resistance to apoptosis. Rather, these findings suggest that FAM60A influences apoptotic competence, potentially through modulation of death receptor signaling or related stress-response pathways. Future studies assessing caspase-8 activation and receptor-specific stimulation will be necessary to clarify the mechanistic contribution of TNFRSF10A to the observed phenotype.

In summary, our work redefines the role of FAM60A as a Sin3/HDAC-associated adaptor that regulates chromatin remodeling and transcriptional control of Hippo signaling. Through its dual-domain structure and indirect SIN3A engagement via HDAC1, FAM60A acts as an essential epigenetic regulator maintaining proliferative homeostasis. Loss of FAM60A unleashes YAP1-driven proliferation and stress resistance, phenotypes reversible through reactivation of the FAM60A–HDAC1–WWC3 axis. Although the present study centers on mechanistic insight at the molecular and cellular levels, in vivo assessment of tumorigenic potential using xenograft models will be an important next step to establish the physiological relevance of FAM60A depletion in breast cancer progression. Taken together, these findings identify the FAM60A–HDAC1–WWC3 module as a promising therapeutic node for reinstating Hippo “off” signals in YAP-addicted cancers.

### Experimental section

#### Materials

Magne® HaloTag® beads (G7281) and SNAP-Capture Magnetic Beads (S9145) were acquired from Promega (Madison, WI) and New England Biolabs (Ipswich, MA), respectively. HaloTag® TMRDirect™ Ligand (G2991) was sourced from Promega. AcTEV™ Protease (12575015) and PreScission Protease (27–0843-01) were obtained from Thermo Fisher Scientific and GE Health Life Sciences, respectively. Micrococcal Nuclease (M0247S) was procured from New England Biolabs. Antibodies used in this study were sourced as follows: RbAp46/RBBP7 (A22274), RBBP4 (A3645), and SIN3A (A21966) from ABclonal, USA; Halo-Tag from Promega, USA (G9211); SAP30 (27679-1-AP) and SUDS3 (25845-1-AP) from Proteintech, USA; FAM60A from MRC PPU, Dundee University, UK (Cat # S381C). Rabbit anti-HaloTag® polyclonal antibody (G9281) was obtained from Promega. Mouse anti-tubulin monoclonal antibody (66031–1-Ig) came from ProteinTech. Goat anti-Mouse IRDye® 680LT (926–68020) and goat anti-Rabbit IRDye® 800CW (926–3211) secondary antibodies were obtained from LI-COR Biosciences. HRP-conjugated Goat Anti-Rabbit IgG (H + L) (SA00001-2) and Goat Anti-Mouse IgG (SA00001-1) secondary antibodies were purchased from Proteintech, USA.

#### Cell culture

HEK293, HEK293T, and MDA‑MB‑231 were purchased from American Type Culture Collection (Manassas, VA, USA) and were maintained in Dulbecco’s Modified Eagle’s Medium (DMEM) supplemented with 10% fetal bovine serum; HEK293 and HEK293T cultures additionally contained 2 mM GlutaMAX. All cell lines were incubated at 37 °C in a humidified incubator with 5% CO₂, and medium was refreshed every 2–3 days.

#### Construction of expression vectors

Expression vectors were generated following previously described methodologies [19, 44]. Briefly, target genes were subcloned into the Halo pcDNA5/FRT vector, specifically between the PacI and PmeI restriction sites. This vector construction was subsequently verified through sequencing. For the production of Halo-tagged constructs expressing FAM60A and SIN3A deletion mutants, the corresponding open reading frames (ORFs) were PCR-amplified and subcloned into the Halo pcDNA5/FRT vector using the Gibson Assembly Cloning Kit [New England Biotechnologies (NEB)]. Specifically, for the creation of a C-terminal Halo-tagged construct of FAM60A (FAM60A-Halo) in the pcDNA5/FRT system, the ORF of FAM60A was amplified using PCR and then subcloned into the pcDNA5FRT C-Halo construct. All plasmid constructs were confirmed by sequencing. The primers used for cloning and PCR are listed in Supplementary Table 5.

#### Preparation of cell lysates

Cells that were confluent or nearly confluent were collected and rinsed twice with ice-cold PBS. Unless otherwise stated, the entire processes were performed at 4 °C (or in an iced environment). Cells were then suspended in a freshly made lysis buffer (containing 20 mM Tris with pH 7.5, 1% Triton, 150 mM NaCl, and protease inhibitors: Aprotinin at 5 mg/L and PMSF at 0.1 mM) and left on ice for half an hour. Then the cells were passed through a 26-gauge syringe several times. This was followed by centrifuging at 14,000 rpm for 15 min at 4 °C.

*Live cell imaging:* For this experiment, HEK293 cells were planted at 20% confluency on glass-bottomed culture dishes (supplied by MatTek, Ashland, MA: (35 mm, No. 2 14-mm diameter glass) and then transiently transfected with Halo-tagged FAM60A/SIN3A and Halo-tagged mutants FAM60A/SIN3A constructs. During growth, affinity-tagged proteins were fluorescently labeled with the Halo-Tag TMRDirect ligand (Promega, WI, USA) according to the manufacturer’s guidelines. Images were captured using a Zeiss LSM 780 confocal microscope, with argon laser excitation at 573–687 nm for TMRDirect. To prevent photobleaching, exposure duration and laser power were tweaked to improve image quality. An alternating excitation mode was implemented to prevent cross-talk among color channels. The HaloTag™ TMRDirect ligand was added to label HaloTag™ proteins at a final concentration of 100 nM, and the cells were incubated overnight at 37 °C in 5% CO_2_. The culture medium was swapped for OptiMEM to minimize background fluorescence before imaging. Cells were stained with Hoeschst dye for 30 min to identify nuclei before imaging.

#### Halo affinity purification for proteomic analysis

HEK293T cells (1 × 10^7^) were cultured in 15-cm plates for 24 h. They were then transfected with Halo-tagged gene constructs using Lipofectamine LTX (Thermo Fisher Scientific, MA, USA). After 48 h, cells were harvested and washed twice with ice-cold PBS. The cells were lysed in 300 μl of mammalian cell lysis buffer (Promega, WI, USA), containing 50 mM Tris-HCl (pH 7.5), 150 mM NaCl, 1% Triton X-100, 0.1% sodium deoxycholate, 0.1 mM benzamidine HCl, 55 μM phenanthroline, 1 mM PMSF, 10 μM bestatin, 5 μM pepstatin A, and 20 μM leupeptin. Lysed cells were passed through a 26-gauge needle 5–7 times and then centrifuged at 14,000 rpm for 12 min at 4 °C. The supernatant (300 μl) was mixed with 700 μl of Tris-buffered saline and centrifuged again at 14,000 rpm for 10 min at 4 °C. The clear supernatant was incubated overnight at 4 °C with 100 μl of prewashed Magne® HaloTag® Beads (Promega, WI, USA). Post-incubation, the beads were washed four times with 750 μl of wash buffer and eluted with a buffer containing 50 mM Tris-HCl (pH 8.0), 0.5 mM EDTA, 0.005 mM DTT, and 2 Units of AcTEV Protease for 2 h at room temperature. Finally, the eluate was passed through a Micro Bio-Spin column to remove any bead residues before proteomic analysis.

#### MudPIT analysis for proteins and protein complexes identification

The procedure for identifying protein complexes using MudPIT analysis has been previously detailed by Banks et al., [44]. To provide a concise summary, proteins purified through trichloroacetic acid precipitation were first subjected to proteolytic digestion with endoproteinase Lys-C, followed by trypsin digestion. This process was carried out overnight at a temperature of 37 °C. Subsequently, a 10-step MudPIT separation technique was utilized, and the digested peptides were directly injected into a linear ion trap mass spectrometer for the collection and identification of spectra. The analysis of peptide mass spectra was performed using the ProLuCID and DTASelect algorithms. We then implemented Contrast and NSAF7 software to arrange the potential affinity-purified proteins based on their respective distributed normalized spectral abundance values. To identify proteins in the experimental samples that were more abundant compared to the control samples, we utilized the QSPEC software. False discovery rates from QSPEC parameters appropriate for multiple comparisons were computed using the Benjamini-Hochberg statistical method. Unless specified otherwise, each experiment was conducted at least three times. All raw mass spectrometry data are accessible as described in Supplementary Table 5.

#### Immunoblotting

Protein lysates were separated on 10% SDS-PAGE gels and then transferred to PVDF membranes (GE Healthcare Life Science). The membranes were blocked in phosphate-buffered saline with Tween 20 (PBST) containing 5% BSA for 1 h at room temperature. Thereafter, membranes were incubated with the respective primary antibodies overnight at 4 °C. After washing three times with Tris-Buffered Saline with Tween 20 (TBST) for 5 min each, the membranes were incubated with horseradish peroxidase-conjugated secondary antibodies at room temperature for 1 h. The membranes were washed three times with TBST. The specific proteins were visualized using the ECL Western blotting system (ThermoFisher, USA). For quantification of western blot band intensity, densitometric analysis was performed using ImageJ software and normalized to the loading control. For SIN3A and mutants’ experiments, membranes were probed with respective primary antibodies, followed by incubation with IRDye® 680LT Goat-anti-Mouse, IRDye® 800CW Goat-anti-Mouse, or IRDye® 800CW Goat-anti-Rabbit secondary antibodies (LI-COR), all diluted 1:10,000. Images of the blots were captured using an Odyssey® CLx imaging system (LI-COR).

#### Gel filtration chromatography

Purified FAM60A protein complexes were isolated from whole cell extracts of HEK293T cells expressing Halo-FAM60A using the Halo affinity purification method. The purified FAM60A was then subjected to size-exclusion chromatography on a Superose 6, 10/300 GL column (Amersham Bioscience) using a buffer composed of 40 mM HEPES (pH 7.9), 350 mM NaCl, 5% glycerol, 0.1% Tween 20, and 1.0 mM DTT. Fractions (500 μl each) were collected to analyze the co-fractionation profiles of Fam60A and its associated proteins, identified through LC-MS/MS (MudPIT). The column calibration was performed using a set of gel filtration markers (Sigma-Aldrich cat. # MWGF1000), including Blue Dextran 2000, thyroglobulin (669 kDa), Ferritin (440 kDa), β-amylase (200 kDa), Alcohol dehydrogenase (150), and bovine serum albumin (66 kDa).

#### Knockout and stable cell generation

For the knockout of the FAM60A, HDAC1, and SUDS3 genes using the CRISPR/Cas9 system, multiple highly specific guide RNAs (gRNAs) targeting three distinct regions of the target genes were designed, synthesized, and inserted into a CRISPR/Cas9 vector [45]. The plasmid construct was then transfected into either HEK293 or MDA-MB-231 cell lines. Following transfection, cells that underwent puromycin selection were selected to establish a homogenous pool of cells with disrupted target genes. The efficiency of the gene knockout was confirmed through Western blot and qRT-PCR analyses. gRNAs used to knock out target genes in this study are listed in Supplementary Table 5.

To stably express FAM60A, stable cell lines were generated as previously described [46]. Briefly, 3×-Flag–tagged FAM60A was cloned into a Tet-on lentiviral vector (Addgene #85400). Lentivirus was produced by transfecting 3 × 10⁶ HEK293T cells in DMEM + 10% FBS with psPAX2 (1.3 pmol), pMD2.G (0.72 pmol), and the FAM60A vector (1.64 pmol) using Lipofectamine LTX & PLUS Reagent (Thermo Fisher Scientific). Viral supernatants were collected at 48 h and 72 h post-transfection, filtered through a 0.45 µm filter, aliquoted, and stored at −80 °C. FAM60A knockout cells were transduced with virus in the presence of 10 µg/mL polybrene; 48 h later, pools were selected with 200 µg/mL G418 (Gibco Geneticin Selective Antibiotic) for 10–12 days. Successful induction of FAM60A-3×Flag was achieved by adding doxycycline (2 μg/mL) and confirmed by Western blot.

#### qRT-PCR

Following the manufacturer’s instructions, total RNA was extracted using the Quick-RNA™ Miniprep Kit, including DNase I treatment to eliminate host DNA contamination. For gene expression assessment, a two-step qRT-PCR approach was used, which targeted both the gene of interest and a housekeeping gene (GAPDH) using SsoAdvanced™ Universal SYBR® Green Supermix (BIO-RAD, CA, USA). First, reverse transcription of 1 µg of RNA was executed with the iScript™ cDNA Synthesis Kit (BIO-RAD, CA, USA). Then a quantitative PCR reaction was performed in a final volume of 20 μl, consisting of 1 × SsoAdvanced™ Universal SYBR® Green Supermix, 500 nM each of forward and reverse primers, 10 ng cDNA, and nuclease-free water. The CFX Opus 96 Real-Time PCR System (Bio-Rad Laboratories, CA, USA) was utilized to quantify relative mRNA expression levels. In the RT-PCR analysis, cDNA was amplified using primers specific to the target genes. The resulting PCR products were then separated on an agarose gel for visualization and confirmation of amplification. Band intensities were subsequently quantified to analyze gene expression with a minimum of three repetitions for all PCR experiments.

#### RNA library preparation, sequencing, and data analysis

Total RNA (RIN ≥ 7) was processed using the MGIEasy Fast RNA Library Prep Set (MGI Tech Co., Ltd., Cat. No. 940-000890-00) following the manufacturer’s instructions. Poly(A)+ mRNA was enriched using the Dynabeads mRNA Purification Kit (Invitrogen, Cat. No. 61006). RNA was fragmented at 94 °C for 6 min to yield ~200 bp inserts, followed by first-strand synthesis and strand-specific second-strand synthesis using dUTP. Subsequent steps included end repair, adapter ligation (MGIEasy UDB Primers Adapter Kit, Cat. No. 1000022800), and PCR amplification. Final libraries were purified with MGIEasy DNA Clean Beads and quality-checked using the Agilent 2100 Bioanalyzer. DNA nanoballs were generated using the DNBSEQ OneStep DNB Make Reagent Kit v2.0 (MGI, Cat. No. 940-000036-00) and sequenced on the DNBSEQ platform with paired-end 150 bp (PE150) reads. Raw sequencing data were processed, which included quality control, filtering out low-quality sequences (Trimmomatic (v0.39)), and aligned and quantified to the reference genome hg38 (Salmon v1.10.1). The R environment (v4.4.3) and the limma R package (v3.62.2) were used for differential gene analysis. A gene was found to be significantly differentially expressed with an absolute log_2_ fold change ≥ 1 and adjusted *p*-value ≤ 0.05.

#### Luciferase reporter assay

We inserted the WWC3 promoter sequence into the pGL4.10[luc2] Vector (Promega Corporation) and confirmed the correct sequence by plasmid sequencing. To examine the impact of FAM60A on the WWC3 promoter, luciferase activity was measured using the Dual-Glo® Luciferase Assay System (Promega Corporation), according to the manufacturer’s protocol. Briefly, 1 × 10^5 FAM60A knockout and FAM60A stably expressed HEK293 cells were seeded in 500 μl complete media in a 24-well plate. At 60–70% confluency, cells were co-transfected with WWC3 promoter containing pGL4.10[luc2] vector (400 ng; firefly luciferase reporter plasmid) and pRL-TK vector (100 ng; Renilla luciferase control plasmid) using Lipofectamine™ LTX with Plus Reagent (catalog# 15338100, Thermo Scientific). After 48 h of transfection, cells were harvested, and both firefly and Renilla luciferase activity were measured in a GloMax 96 Microplate Luminometer (Promega Corporation). We transfected cells with empty pGL4.10[luc2] Vector and/or pRL-TK plasmid to measure the background luciferase activity. Relative luciferase activity was calculated by normalizing firefly to Renilla luciferase activity, and fold changes in luciferase activity were calculated accordingly. Statistical significance was evaluated using Student’s t-test.

#### ChIP-qPCR

A ChIP-qPCR was performed as described previously [47] with some modifications. Briefly, FAM60A stably expressing HEK293 cells were cultured in complete medium containing 2 μg/ml doxycycline (we inserted Flag-tagged FAM60A under the Tet-on promoter). Similarly, we cultured HEK293 wild-type and FAM60A-knockout cells to assess the gene-activation markers H3K4me3 and H3K27ac on WWC3 promoter and enhancer regions. Cells were cross-linked with 1% formaldehyde for 10 min at room temperature, followed by quenching with 125 mM glycine for 5 min. After harvesting, cells were lysed in IP buffer (150 mM NaCl, 50 mM Tris-HCl (pH 7.5), 5 mM EDTA, NP-40 (0.5% vol/vol), Triton X-100 (1.0% vol/vol), including protease and phosphatase inhibitors. After cell lysis, nuclei were sonicated in 500 μl IP buffer under the following conditions: seven rounds of 15 1-s pulses at 50% power output. To immunoprecipitate FAM60A-bound DNA, sonicated chromatins from 10 million cells were incubated overnight at 4 °C with 4 μg anti-FLAG primary antibody (66008-4, Proteintech®) or mouse IgG (sc-2025, Santa Cruz). In addition, to immunoprecipitate H3K4me3 and H3K27ac-marked DNA, an equal amount of sonicated chromatin was incubated overnight at 4 °C with 4 μg of anti-H3K4me3 (ab4729, abcam) or anti-H3K27ac primary antibody (ab8580, abcam) or normal rabbit IgG (catalog#2729, Cell Signaling Technology). The antibody-antigen complexes were precipitated with Dynabeads™ Protein G magnetic beads (catalog#10007D, Invitrogen^TM^) for 4 h at 4 °C. After four washes, chromatin-antigen complexes were eluted in 100 mM NaHCO3/1% SDS at 65 °C for 15 min. Eluents were reverse-cross-linked with 200 mM NaCl at 65 °C overnight. DNA was extracted using a column-based DNA purification kit after the eluted chromatin-antigen complex was treated with proteinase K. To confirm the FAM60A or H3K4me3 and H3K27ac binding at the WWC3 promoter and enhancer regions, we performed qPCR from the ChIP sample and ChIP IgG in a CFX Opus 96 Real-Time PCR System (Bio-Rad Laboratories, CA, USA). We calculated fold enrichment using the formula: 2^[Ct(IgG) – Ct(sample)]. Statistical analyses were performed using Student’s t-test. Primers for the ChIP-qPCR analysis were designed using PrimerQuest Tool (https://www.idtdna.com/PrimerQuest/Home/Index) and listed in Supplementary Table 5.

#### Apoptosis assay

Apoptotic cell death was quantified using the Dead Cell Apoptosis Kit with Annexin V–FITC and propidium iodide (PI) (Invitrogen, Cat. No. V13242) exactly as per the manufacturer’s instructions. HEK293 (WT, FAM60A-KO, FAM60A-Res) and MDA-MB-231 (WT, FAM60A-KO, FAM60A-Res) cells were seeded at 2 × 10^5^ cells per well in 6-well plates and allowed to adhere overnight at 37 °C in a 5% CO₂ incubator. Following treatment with 10 μM metformin or or 5 μM doxorubicin for 24 h as a positive control, cells were harvested by trypsinization, washed twice with ice-cold PBS, and resuspended in 1× annexin-binding buffer at ~1 × 10^6^ cells/mL. Aliquots of 100 µL cell suspension were stained with 5 µL FITC Annexin V and 1 µL of the 100 µg/mL PI working solution for 15 min at room temperature in the dark; afterward, 400 µL of 1× annexin-binding buffer was added, samples were kept on ice, and data were acquired immediately on a BD LSRFortessa flow cytometer (≥10,000 events per sample). Compensation controls included unstained cells, Annexin V–FITC only, and PI only. Data was acquired for at least 10,000 events per sample and analyzed with FlowJo v10.

#### Cell-cycle analysis

HEK293 (WT, FAM60A-KO, FAM60A-Res) and MDA-MB-231 (WT, FAM60A-KO, FAM60A-Res) cells were seeded at 2 × 10^5^ cells per well in 6-well plates and allowed to adhere overnight at 37 °C in a humidified 5% CO₂ incubator. After trypsinization and two washes with ice-cold PBS, cell pellets were gently resuspended in 500 µL of ice-cold PBS; ice-cold 100% ethanol was added dropwise with gentle vortexing to reach a final concentration of 70%, and samples were fixed on ice for at least 2 h. Fixed cells were pelleted at 500 × *g* for 5 min at 4 °C, washed twice with PBS to remove residual ethanol, and then resuspended in 500 µL staining buffer (PBS with 100 µg/mL RNase A and 50 µg/mL propidium iodide). Tubes were wrapped in foil and incubated overnight at 4 °C in the dark before acquiring at least 20,000 events per sample on a BD LSRFortessa flow cytometer. Cell-cycle phase distribution (G₀/G₁, S, G₂/M) was analyzed using the Watson pragmatic model in FlowJo v10.

#### Subcellular protein fractionation

Subcellular protein fractionation was performed using the Subcellular Protein Fractionation Kit for Cultured Cells (Thermo Scientific, Waltham, MA; Cat. No. 78840) according to instructions provided by the manufacturer. HEK293 control and FAM60A knockout cells were grown to ~90% confluence, harvested by scraping, and washed twice with ice‑cold phosphate‑buffered saline. Cell pellets were resuspended in Cytoplasmic Extraction Buffer supplemented with 1× protease inhibitor cocktail and incubated on ice for 10 min. Samples were centrifuged at 500 × *g* for 5 min at 4 °C, and the supernatant was collected as the cytosolic fraction. The remaining pellet was incubated with Membrane Extraction Buffer containing 1× protease inhibitor cocktail for 10 min on ice, then centrifuged at 3000 × *g* for 5 min at 4 °C to yield the membrane fraction. Nuclear proteins were extracted from the final pellet using Nuclear Extraction Buffer. Protein concentrations were determined by bicinchoninic acid assay (Pierce™ BCA Protein Assay Kit, Thermo Scientific, Cat. No. 23225). Fraction purity was confirmed by immunoblotting with GAPDH (cytosolic/membrane marker) and Histone H3 (nuclear marker). Equal amounts of each fraction were separated by SDS–PAGE and probed with antibodies against YAP1, TAZ, and FAM60A.

#### Immunoprecipitation of FAM60A

HEK293 cells were cultured in 150-mm dishes. Before harvesting, cells were washed twice with ice-cold PBS, scraped, and pelleted by centrifugation at 1000 × *g* for 4 min at 4 °C. For lysate preparation, the pellet was resuspended in Mammalian Lysis Buffer supplemented with 1× protease inhibitors and incubated on ice for 30 min. Cells were disrupted by passing the suspension through a 26-gauge needle 10 times, followed by centrifugation at 16,000 × *g* for 15 min at 4 °C to obtain clarified lysate. Co-immunoprecipitation was performed using the Dynabeads™ Protein G Immunoprecipitation Kit (Catalog #10007D; Invitrogen) according to the manufacturer’s protocol. Clarified lysate was incubated overnight at 4 °C with 4 µg sheep anti-FAM60A antibody (Catalog #S381C; MRC PPU Reagents and Services, University of Dundee) or sheep IgG control. Antibody–antigen complexes were then incubated with Protein G–coupled magnetic beads for 4 h at 4 °C with rotation. Beads were washed four times to remove nonspecific proteins, and bound complexes were eluted in Laemmli sample buffer by incubation at 95 °C for 10 min. Ten percent of each immunoprecipitate was analyzed by Western blot to assess interactions between FAM60A and SIN3A, HDAC1, HDAC2, and SUDS3.

#### YAP1 knockdown using siRNA and cell proliferation assay

Two human YAP1-targeting siRNAs (J-012200-05-0002 and J-012200-07-0002; Dharmacon™ reagents) were used for gene silencing. FAM60A-knockout HEK293 cells (6 × 10⁵ per well) were seeded into 6-well plates and transfected the following day with 40 nM of a single YAP1 siRNA using Lipofectamine™ RNAiMAX (Invitrogen), following the manufacturer’s instructions. At 24 h post-transfection, a fraction of the cells was reseeded into 96-well plates to assess proliferation, while the remaining cells were maintained for analysis of knockdown efficiency. Cell proliferation was quantified at 48 h and 72 h post-transfection using the Premix WST-1 Cell Proliferation Assay Kit (Takara Bio USA). Statistical comparisons were performed using unpaired t-tests in GraphPad Prism (v8.0.2). At 72 h, cells were harvested for Western blotting to confirm YAP1 knockdown.

#### Statistical analysis

For the analysis of quantitative PCR (qPCR) data involving multiple comparisons, we employed a Student’s t-test or one-way analysis of variance (ANOVA) followed by a Newman-Keuls post hoc test where appropriate. This statistical analysis was conducted using GraphPad Prism software, version 7 and 10 (GraphPad Software, San Diego, CA, USA). We presented the results as means ± standard deviation (SD), with a sample size of n ≥ 3, unless specified otherwise. A *p*-value of 0.05 or less (*P* ≤ 0.05) was considered to indicate statistical significance.

## Supplementary information

Supplementary Figure Legend

Supplementary Figure 1

Supplementary Figure 2

Supplementary Figure 3

Supplementary Figure 4

Supplementary Figure 5

Supplementary Figure 6

Supplementary Figure 7

Supplementary Table 1

Supplementary Table 2

Supplementary Table 3

Supplementary Table 4

Supplementary Table 5

## Data Availability

All data supporting the paper’s conclusions are available in the paper and its Supplementary Materials. Mass spectrometry datasets generated in this study are deposited in the Massive repository and ProteomeXchange (https://massive.ucsd.edu and https://proteomecentral.proteomexchange.org/ui; identifiers in Supplementary Table S5) and RNA-seq data in GEO (Accession: GSE253631; http://www.ncbi.nlm.nih.gov/geo/). Additional data related to this paper may be requested from the authors. Materials inquiry requests should be made to msmiah@uams.edu.
