# Supplementary material for: Loss of FAM60A disrupts Sin3/HDAC control of the Hippo signaling and promotes oncogenic YAP1 activation
Source: Cell Death Dis. 2026 Apr 27;17(1):560. doi: 10.1038/s41419-026-08778-y (PMC13254352; doi:10.1038/s41419-026-08778-y)
Supplement: Supplementary file 1 — Supplementary Figure Legend [file 41419_2026_8778_MOESM1_ESM.docx]

**Supplementary Figure legend:**

**Suppl. Fig. S1: Domain-specific protein-protein interaction of the SIN3A Protein. A** schematic diagram illustrating various deletions in the *SIN3A* gene. **B** Halo-tagged SIN3A deletion mutants were transfected into HEK293T cells. Halo-Tag TMRDirect fluorescent ligand (red) was used to label Halo-Tag proteins; DNA was stained with Hoechst dye (blue). **C-D** Halo-SIN3A deletion mutants were ectopically expressed in HEK293T cells, followed by Halo affinity purification, and the purified proteins were subjected to proteomics analysis (log2FC ≥ 2, FDR < 0.05) and visualized in a heatmap (green and black) (**D)** identified SIN3A domain-specific interaction with core subunits has been visualized in a heatmap (grey indicates no interaction).

**Suppl. Fig. S2: Cross-species similarity of FAM60A amino acid sequences. A** The FAM60A protein sequence was analyzed using the standard protein BLAST tool on the NCBI BLAST website, aligning the query sequence with those in a specified target database. **B** A schematic diagram depicts various deletions in the FAM60A gene. Halo-tagged FAM60A mutants (FAM60A_Δ1 (1-148), FAM60A_Δ2 (1-95), FAM60A_Δ3 (95-148), FAM60A_Δ* (95-221), and FAM60A_Δ4 (149-221, spliced variants) were transfected into HEK293 cells, and post-halo affinity purification, the proteins were resolved on SDS-PAGE and visualized via silver staining.

**Suppl. Fig. S3: FAM60A employs various domains to facilitate interactions with different proteins. A** full-length FAM60A, along with its deletion mutants FAM60A_Δ1 (1-148) and FAM60A_Δ4 (149-221, spliced variants), were Halo-tagged and transfected into HEK 293 cells. Post-transfection, Halo affinity purification was performed, and the proteins underwent proteomics analysis (log2FC ≥ 2, FDR < 0.05), followed by visualization in an interaction network via Cytoscape. **B** Halo-tagged FAM60A was transfected into HEK 293 cells, followed by Halo affinity purification. The purified proteins were analyzed through immunoblotting using various antibodies, with Halo and GAPDH serving as loading controls. **C** the conserved motif in FAM60A's C-terminus (FAM60A_Δ4 149-221: spliced variant) was identified using the standard protein blast tool on the NCBI BLAST website. The conserved motif is highlighted and marked with asterisks at the bottom.

**Suppl. Fig. S4: Crosstalk among major signaling pathways**. KEGG analysis of differentially expressed genes identified by RNA-seq (FDR ≤ 0.05) between FAM60A‑knockout and control HEK293 cells uncovered extensive crosstalk among Hippo, TGF-β, and Wnt pathways. The results revealed extensive crosstalk among major signaling pathways, highlighting how these pathways jointly regulate anti-apoptotic and pro-proliferative genes.

**Suppl. Fig. S5: FAM60A-dependent regulation of WWC3. A** ChIP-Seq analyses from two separate studies show that FAM60A’s binding partner Sin3A binds in the enhancer (target region: chromosome X: 10,011,376-10,011,655) and promoter (target region: chromosome X: 10,014,280-10,015,535) region of WWC3. **B** The expression of Halo-FAM60A and Halo-FAM60A motif-3 was confirmed by immunoblot using antibodies against FAM60A, and GAPDH as a loading control. **C** MDA-MB-231 control and FAM60A-knockout cells were fixed and processed for immunohistochemical staining using antibodies against YAP1. **D** Protein levels of CTGF and CYR61 were examined in control and FAM60A-knockout cells by immunoblotting using specific antibodies, with GAPDH as a loading control.

**Suppl. Fig. S6: FAM60A regulates cell‑cycle and proliferation through Hippo signaling. A** FAM60A protein expression in HEK 293 control cells, FAM60A knockout cells generated via CRISPR-Cas9, and FAM60A reintroduced in the knockout cells, was determined using immunoblotting and probed with anti-FAM60A antibody and GAPDH as a loading control. **B** Proliferation analysis of HEK 293 control cells, FAM60A knockout cells, and Flag-WWC3 overexpressed in the knockout cells. Cell proliferation was quantified using the WST-1 assay (***p ≤ 0.001, n=3; ns: not significant). The expression of FLAG-WWC3 was confirmed by immunoblot using antibodies against FLAG, and GAPDH as a loading control. **C** FAM60A-knockout HEK293 cells were transfected with two independent siRNAs targeting YAP1. YAP1 protein levels were assessed by immunoblotting using an anti-YAP1 antibody, with GAPDH as a loading control. Cell proliferation was quantified using the WST-1 assay (***p ≤ 0.001, n=3). **D** Cell‑cycle profiles of HEK293 control, FAM60A‑knockout, and FAM60A reintroduced cells determined by propidium iodide‑based flow cytometry; representative DNA‑content histogram shown (G1, S, G2; mean ± SD, n = 3). **E** FAM60A protein expression in MDA-MB-231 cells, FAM60A knockout cells generated via CRISPR-Cas9, and FAM60A reintroduced in the knockout cells, were determined using immunoblotting and probed with anti-FAM60A antibody and GAPDH as a loading control. **F** Cell‑cycle profiles of MDA-MB-231 control, FAM60A‑knockout, and FAM60A reintroduced cells determined by propidium iodide‑based flow cytometry; representative histogram shown (G1, S, G2; n = 3).

**Suppl. Fig. S7: Apoptosis induced by chemotherapeutic and metabolic stress agents in FAM60A-manipulated HEK293 and MDA-MB-231 cells. A** and **B**, Apoptosis in control, FAM60A-knockout, and FAM60A-reintroduced HEK293 and MDA-MB-231 cells following 24 h treatment with 10 µM metformin, assessed by Annexin V/propidium iodide staining and analyzed by flow cytometry; representative cytometric profiles are shown (n = 3). **C** and **D**, Apoptosis in control, FAM60A-knockout, and FAM60A-reintroduced HEK293 and MDA-MB-231 cells following 24 h treatment with 5 µM doxorubicin, assessed by Annexin V/propidium iodide staining and analyzed by flow cytometry; representative cytometric profiles are shown (n = 3). Mean ± SD (n=3); ***P* ≤0.01, **P* ≤0.05. **ns**: not significant. **WT**: wild type; **KO**: knockout; **Res**: rescue with FAM60A in KO cells.
