# Supplementary figures and images for: Loss of FAM60A disrupts Sin3/HDAC control of the Hippo signaling and promotes oncogenic YAP1 activation
Source: Cell Death Dis. 2026 Apr 27;17(1):560. doi: 10.1038/s41419-026-08778-y (PMC13254352; doi:10.1038/s41419-026-08778-y)

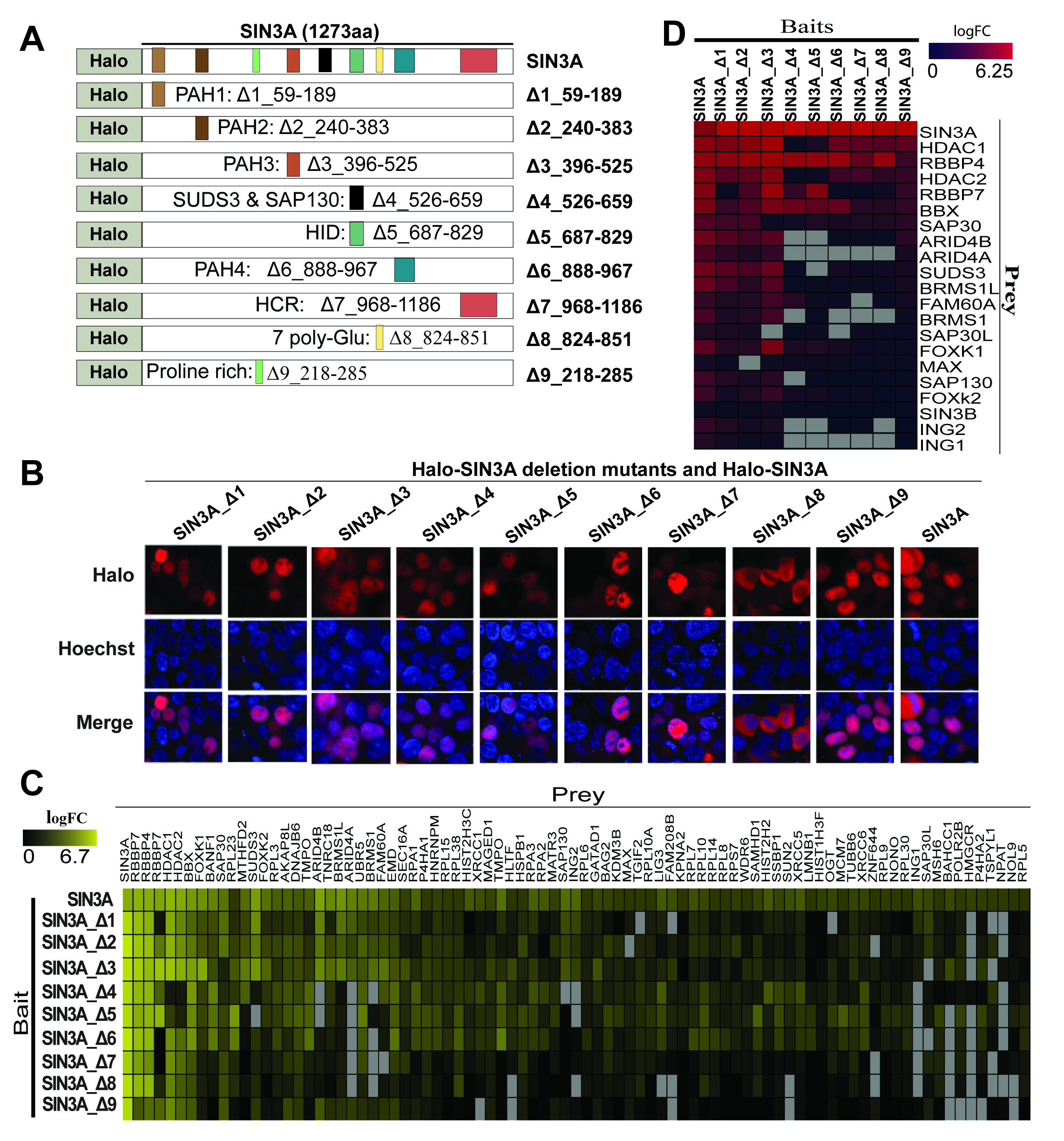

Supplement: Supplementary file 2 — Supplementary Figure 1 [file 41419_2026_8778_MOESM2_ESM.tif]

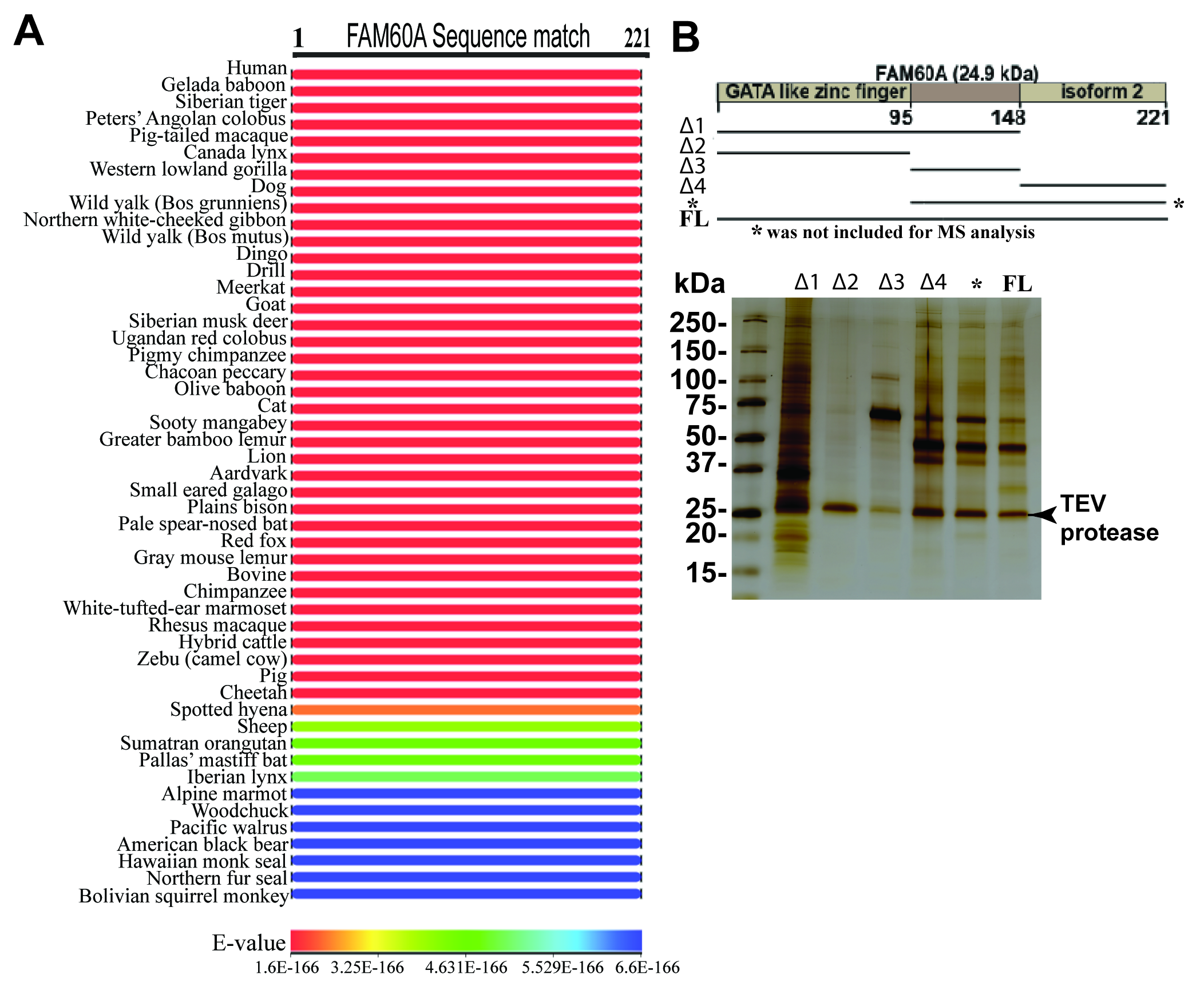

Supplement: Supplementary file 3 — Supplementary Figure 2 [file 41419_2026_8778_MOESM3_ESM.tif]

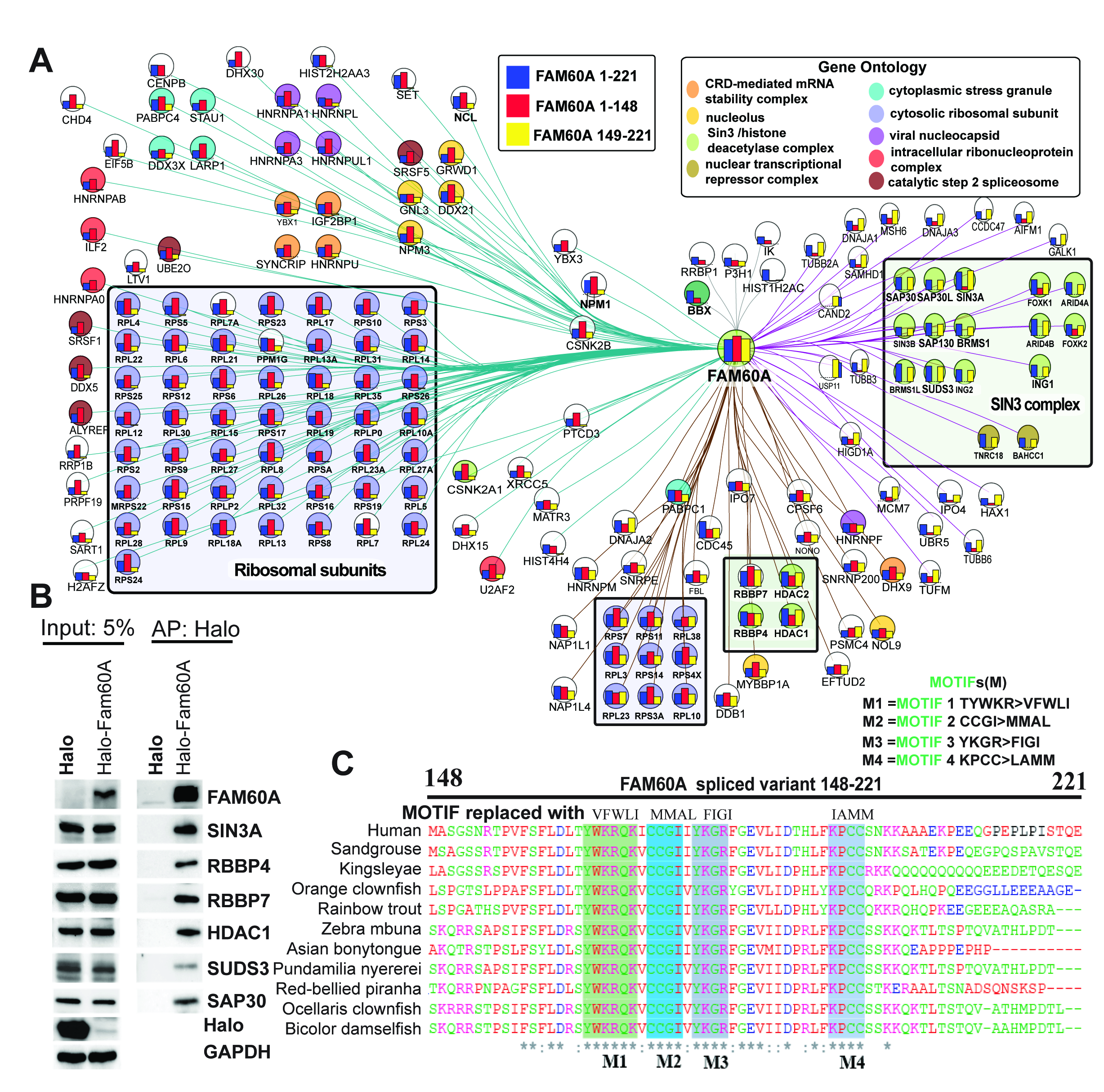

Supplement: Supplementary file 4 — Supplementary Figure 3 [file 41419_2026_8778_MOESM4_ESM.tif]

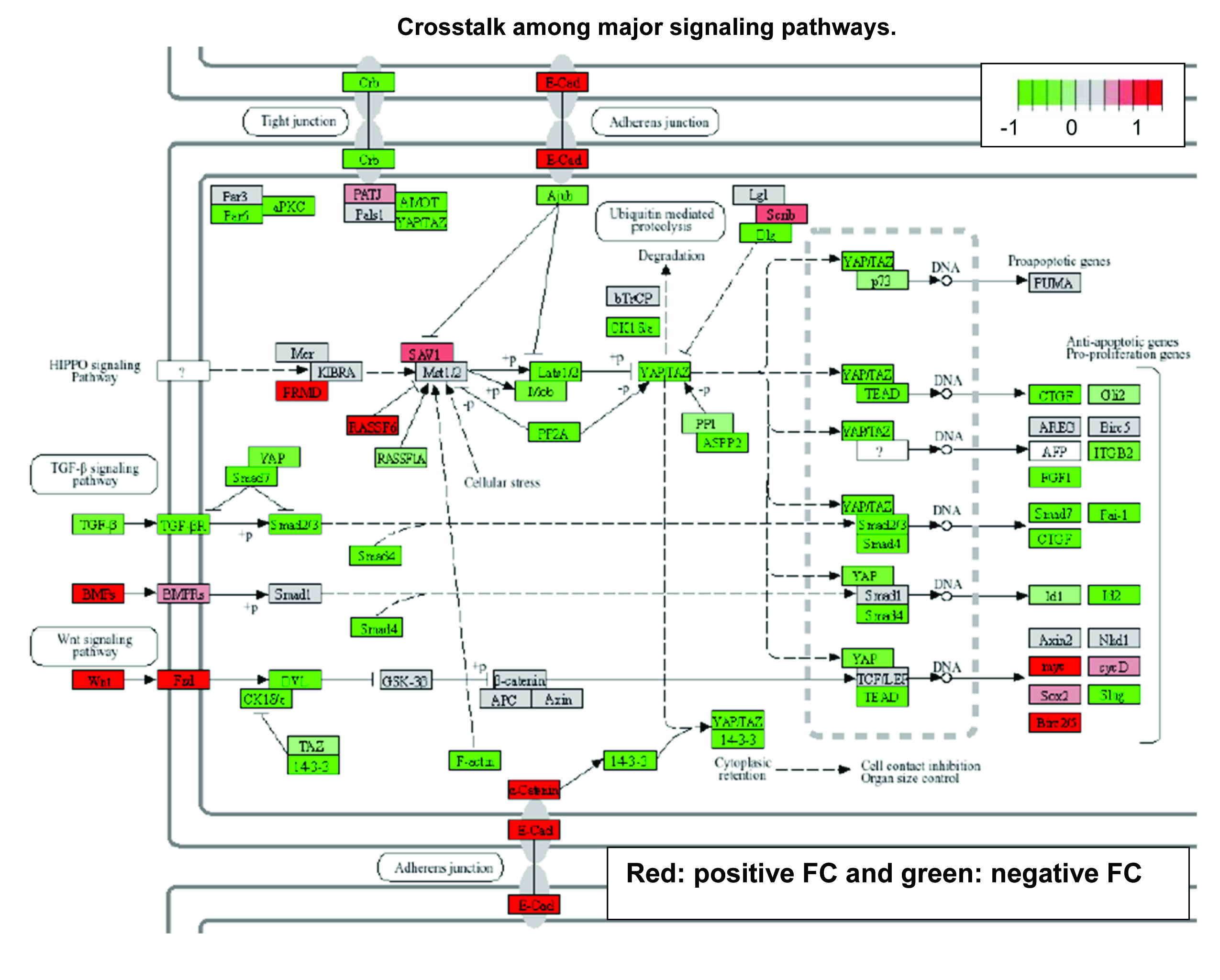

Supplement: Supplementary file 5 — Supplementary Figure 4 [file 41419_2026_8778_MOESM5_ESM.tif]

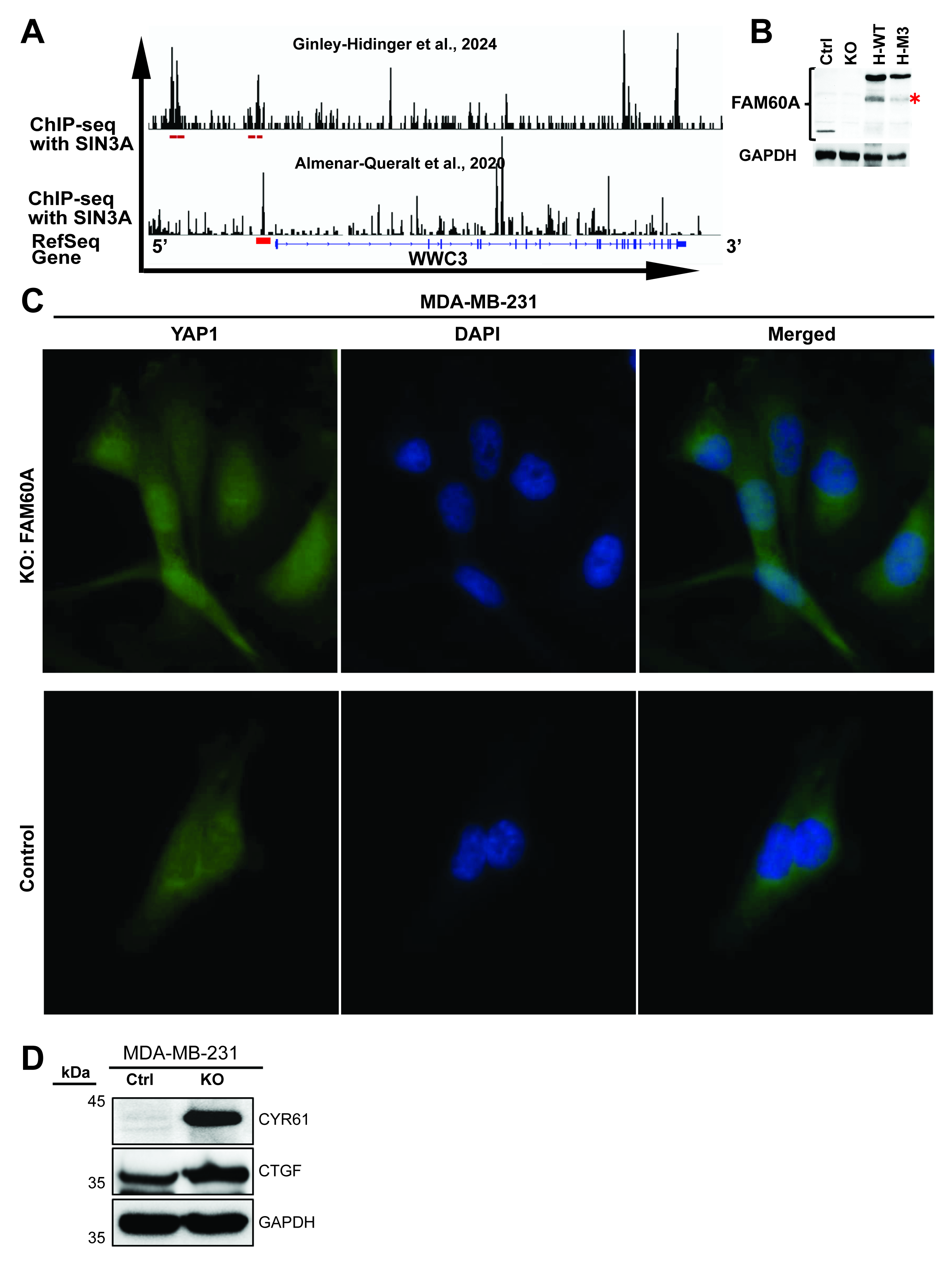

Supplement: Supplementary file 6 — Supplementary Figure 5 [file 41419_2026_8778_MOESM6_ESM.tif]

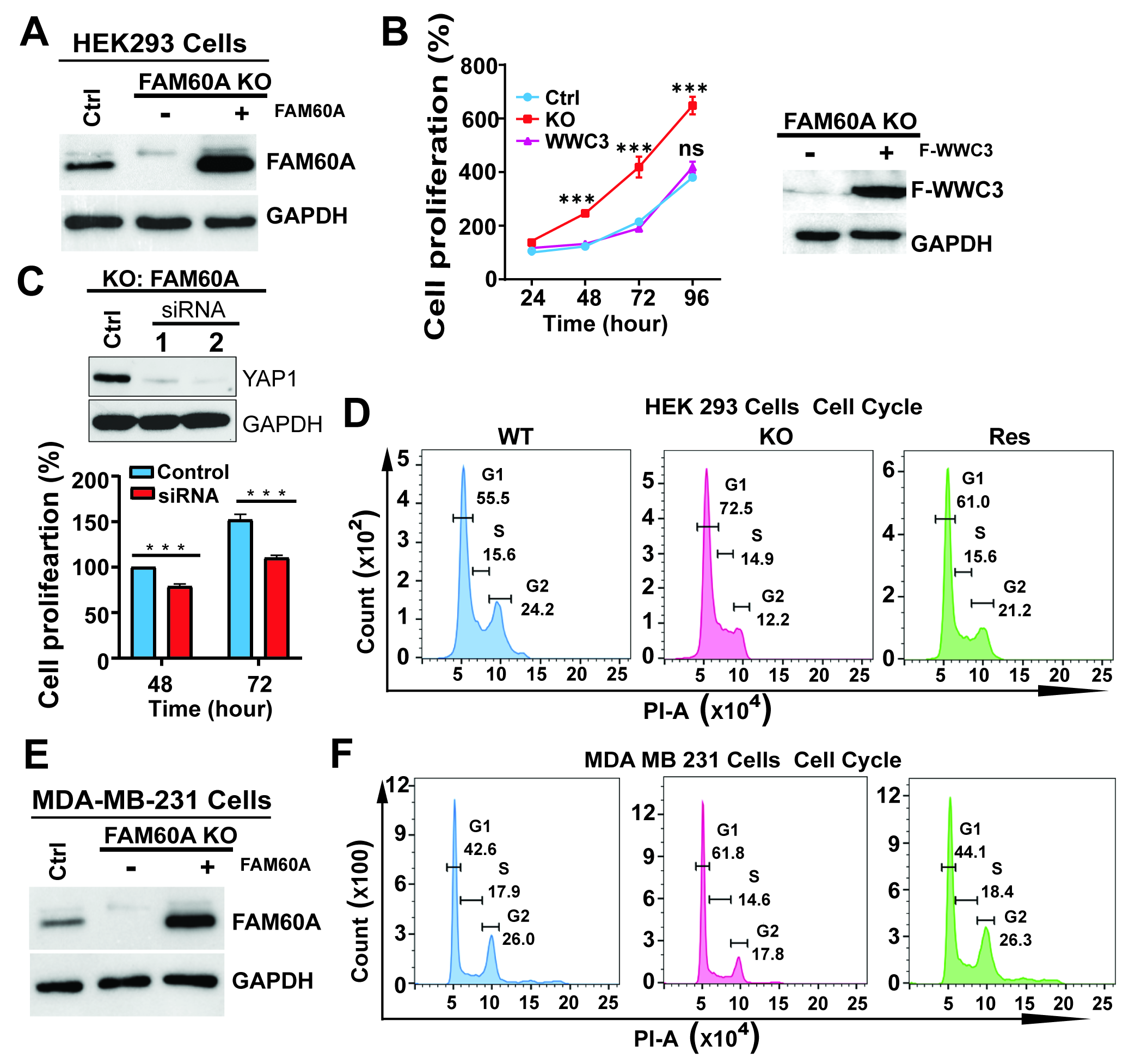

Supplement: Supplementary file 7 — Supplementary Figure 6 [file 41419_2026_8778_MOESM7_ESM.tif]

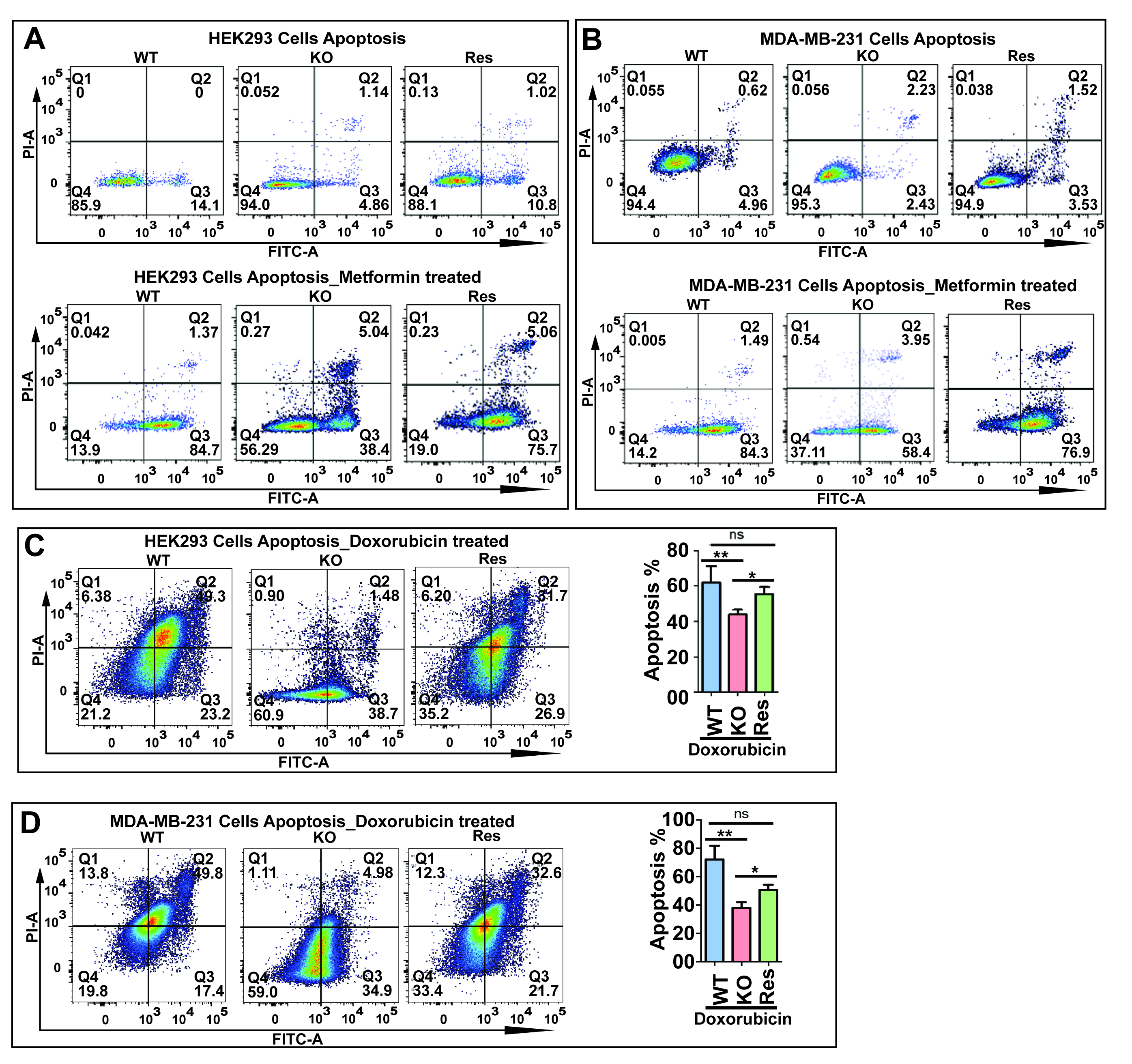

Supplement: Supplementary file 8 — Supplementary Figure 7 [file 41419_2026_8778_MOESM8_ESM.tif]
